# Role of Structural Modifications in Peptidomimetic Compounds as Potential Antimicrobial Agents against *Staphylococcus aureus* and *Streptococcus pyogenes*: Balancing Bioavailability, Safety, and Antimicrobial Activity

**DOI:** 10.1021/acsomega.5c03775

**Published:** 2025-07-22

**Authors:** Maria Dzierżyńska, Justyna Sawicka, Katarzyna Łada, Agnieszka Gajewicz-Skretna, Milena Deptuła, Alexey Chernobrovkin, Aneta Pogorzelska, Anders Grubb, Roman A. Zubarev, Michał Pikuła, Franciszek Kasprzykowski, Sylwia Rodziewicz-Motowidło

**Affiliations:** † Department of Biomedical Chemistry, Faculty of Chemistry, University of Gdańsk, Wita Stwosza 63, 80-308 Gdansk, Poland; ‡ Department of Molecular Biotechnology, Mossakowski Medical Research Institute Polish Academy of Science, Wita Stwosza 63, 80-308 Gdansk, Poland; § Department of Physical Chemistry, Faculty of Chemistry, Gdańsk University of Technology, Narutowicza 11/12, 80-233 Gdansk, Poland; ∥ Laboratory of Tissue Engineering and Regenerative Medicine, Division of Embryology, Medical University of Gdansk, Dębinki 1, 80-211 Gdansk, Poland; ⊥ Department of Medical Biochemistry and Biophysics, 27106 Karolinska Institutet, Solnavägen 9, 17177 Stockholm, Sweden; # Pelago Bioscience AB, Scheelesväg 1, 17165 Solna, Sweden; ∇ Department of Organic Chemistry, Faculty of Pharmacy, Medical University of Gdańsk, Hallera 107, 80-416 Gdansk, Poland; ○ Department of Clinical Chemistry, Skåne University Hospital, Lund University, Klinikgatan 19, 22185 Lund, Sweden

## Abstract

The emergence of drug-resistant Gram-positive pathogens, particularly*Staphylococcus aureus*and*Streptococcus pyogenes*, has driven the need for novel antimicrobial agents. This study explores 21 newly synthesized peptidomimetic analogues of cystatin C *N*-terminal fragment, designed to enhance bioactivity, solubility, and safety. These compounds were evaluated for antimicrobial potency, cytotoxicity, pro-proliferative effects, and pharmacokinetic properties. Key findings indicate that analogues A-192 and A-164 exhibited the strongest antimicrobial effects against *S. aureus* and *S. pyogenes*. Most compounds were inactive against Gram-negative bacteria. Cytotoxicity profiling identified several derivatives with low to moderate toxicity and favorable pro-proliferative effects at specific concentrations. Stability tests confirmed the robustness in aqueous and plasma environments. Computational absorption, distribution, metabolism, excretion, and toxicity (ADMET) modeling revealed low gastrointestinal absorption, but favorable parameters for topical applications. Exploratory analyses (principal component analysis (PCA) and two-way hierarchical cluster analysis (2D-HCA)) linked structural features—such as branching, molecular weight, and solubility, with biological activity. These results support the potential of structurally optimized peptidomimetics as targeted, topical therapeutics for Gram-positive infections and provide a rationale for further preclinical development.

## Graphical abstract

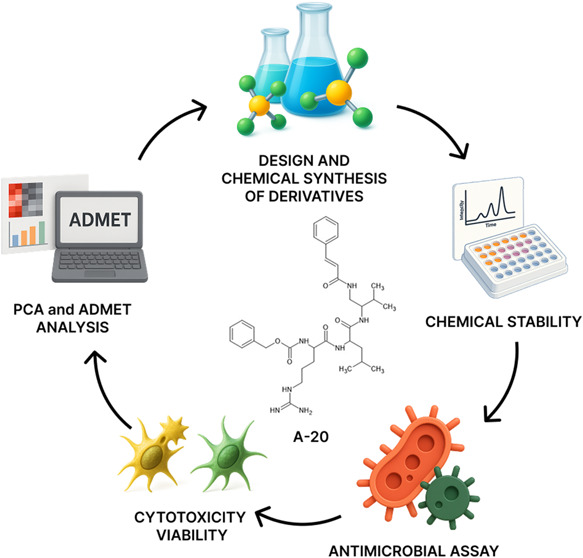

## Introduction

Antibiotics have revolutionized the treatment of bacterial infections since their discovery. However, shortly after the discovery of the first antibiotic, Alexander Fleming warned that its extensive use may result in bacterial resistance.^1^ Unfortunately, it appears that his predictions align with current developments. Decades of antibiotic misuse in medicine and overuse in agriculture and animal farms have caused the appearance of bacterial strains resistant to most, if not all, known antibiotics.^2–4^ The increasing prevalence of ESKAPE bacteria—i.e., *Enterococcus faecium*, *Staphylococcus aureus*, *Klebsiella pneumoniae*, *Acinetobacter baumannii*, *Pseudomonas aeruginosa*, and *Enterobacter* spp.—is a growing concern, as it may lead to infections that are difficult or even impossible to treat.^5^ The emergence of the COVID-19 pandemic has substantially contributed to the prevailing circumstances.^6–8^ Therefore, the development of new-generation antimicrobial compounds is sorely needed if we wish to cope with infectious diseases.^9^ Most commonly used antibiotics were developed more than 30 years ago. Despite great advances in biotechnology, very few new antibacterial drugs have been introduced to common pharmaceutical use since then.^10^ However, the rapid development of artificial intelligence is opening new possibilities for drug discovery, including the design of novel antibiotics, which could lead to groundbreaking solutions in the search for effective antimicrobial agents.

Among the compounds with antibacterial activity, antimicrobial peptides (AMPs) have garnered attention for their potent activity against a wide range of pathogens. Their antimicrobial mechanism of action is primarily associated with targeting cell membranes, due to their cationic nature.^11,12^ However, there is evidence that peptides may act intracellularly, after the cell-penetrating step, by targeting nucleic acids, proteins, proteases, and cell division mechanics.^12–14^

In the class of antimicrobial molecules, human cystatin C may also be included. It is a small protein whose main purpose seems to be to protect tissues from excessive lysosomal cysteine proteases. Human cystatin C also has antibacterial and antiviral effects. Combined with the fact that it is present in all tissues and body fluids, this makes it part of the first line of the human immune system.^15–18^

In addition to peptides and proteins with antibacterial activity, a large group is constituted by small antimicrobial peptidomimetics, which offer an innovative approach combining the desirable properties of the peptide design with the structural motif of natural peptides, paving the way toward development of novel therapeutic agents.^19^ Peptidomimetics designed based on the *N*-terminal structure of cystatin C has been studied by our group for some time.^20–23^ In this article, we present twenty-one newly synthesized analogues of one of the most potent peptidomimetic in this series, Cystapep 1 (A-20). Given its significant antimicrobial potential, we aimed to optimize its structure to reduce the minimum inhibitory concentration (MIC) values against methicillin-resistant*S. aureus* and other Gram-positive bacteria, while maintaining low cytotoxicity and enhancing solubility. Additionally, due to the skin-colonizing nature of these bacteria, we evaluated the pro-proliferative effect of these peptidomimetics on skin cells. Along with the experimental data, we employed exploratory data analysis and computed pharmacokinetic parameters, including absorption, distribution, metabolism, excretion, and toxicity (ADMET), to aid in optimization and assessment of the compounds’ potential for future application.

## Materials and Methods

### Synthesis

The synthesis of A-20 peptidomimetic has previously been described.^22^ Syntheses of peptidomimetics were carried out by classical, in solution method using (2*S*)-*N* ^2^-Boc-1,2-diamines (specifically (2*S*)-*N* ^2^-Boc-1,2-diamino-3-methylbutane and ((2*S*)-*N* ^2^-Boc-1,2-diaminobutane)). The latter were obtained from Boc-Val-OH and Boc-Abu-OH in accordance with the general procedure presented in Scheme 1. The amino acid was reduced to the corresponding alcohol using NaAlH_2_(OCH_2_CH_2_OCH_3_)_2_ as the reducing agent (as described previously^24^). The resulting alcohol was then converted to the mesylate to facilitate nucleophilic substitution with the azide. Finally, conversion to the amine hydrochloride was accomplished by hydrogenolysis.^20^ The corresponding diamine residues, introduced using these reagents, are given in Table 1 as −NH–CH­(iPr)–CH_2_–NH– and −NH–CH­(CH_2_–CH_3_)–CH_2_–NH–, respectively. All amino acids were in the L form. The elongation from lysine to homoarginine (described as Har in Table 1) in A-134, A-192, A-193, and A-194 was obtained by using 3,5-dimethyl-1-pyrazolylformaminidium nitrate, detailed in a previous article.^23^ The homologation of Boc-Val-OH to Boc-βLeu-OH for A-152 synthesis was prepared via an Arndt–Eistert synthesis using isobutyl chloroformate and diazomethane to obtain a diazoketone, as represented in Scheme 2. Wolff rearrangement of the diazoketone to a carboxylic acid was achieved with water in the presence of Ag_2_O as a catalyst.^25,26^ The final product was coupled with amino acids and is described as −NH–CH­(iPr)–CH_2_–CO– in Table 1. *Z*(*o*-Me)-Arg-OH for A-210 was synthesized using 2-methylbenzyl alcohol and triphosgene in the presence of pyridine and then coupled with arginine. Other substrates were commercially purchased. For all compounds, *N*,*N*′-dicyclohexylcarbodiimide (DCC) (Sigma-Aldrich, USA) was used as a coupling agent, and hydroxybenzotriazole (HOBt) (Merck Millipore, USA) was used as a racemization suppressor. The Boc-protecting group was cleaved using anhydrous 4 M HCl in dioxane. In the case of A-198, the benzyloxycarbonyl (*Z*) protecting group was removed by hydrogenolysis, simultaneously with the reduction of the double bond of cinnamic acid. Crude peptidomimetics were purified with a high-performance liquid chromatography (HPLC) system equipped with a Kromasil C8 (100 Å, 5 μm, 21.2 × 250 mm^2^) column using isocratic modes. The molecular masses were determined using a liquid chromatography-mass spectrometry (LC-MS) system (Shimadzu, Japan) and purity (above 95%) was ascertained by HPLC analysis in full gradient mode.

**Scheme 1:**
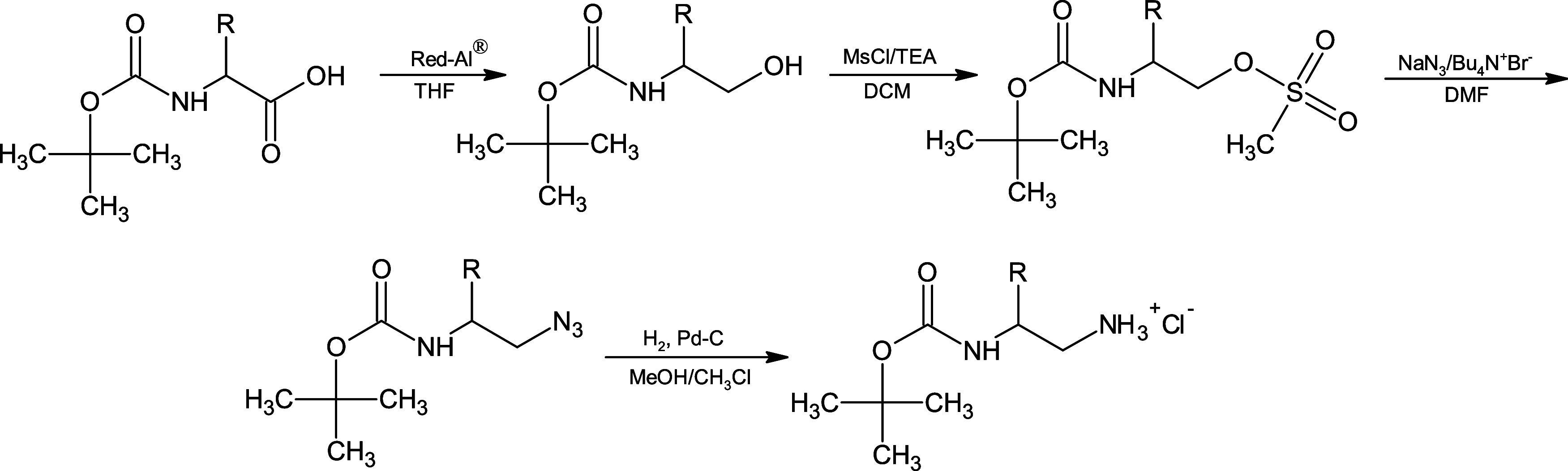
General Procedure for Preparation (2*S*)*-N* ^2^-Boc-1,2-diamines

**Scheme 2:**
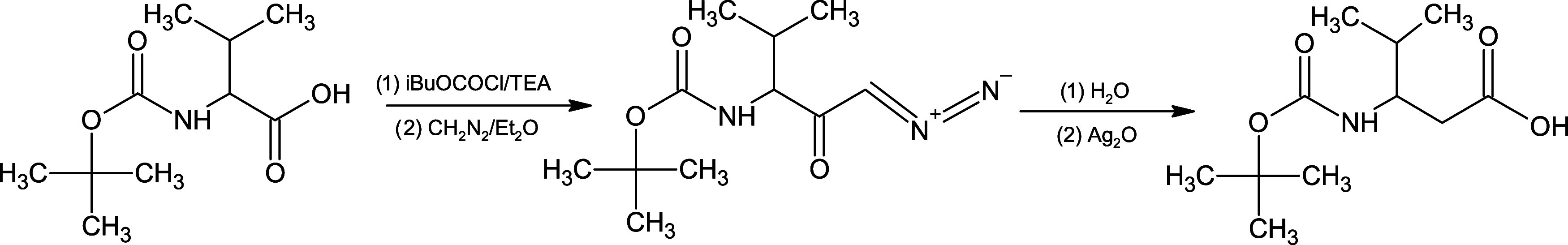
Synthesis of Boc-βLeu-OH for Compound A-152

**Table 1:** Synthesized New Compounds with Potential Antimicrobial Activity^a^

no	code	P1	P2	P3	P4
0	A-20	*Z*-Arg-	-Leu-	–NH–CH(iPr)–CH_2_–NH–	–Cin
1	A-134	*Z*-Har	-Leu-	–NH–CH(iPr)–CH_2_–NH–	–Cin
2	A-152	*Z*-Arg-	-Leu-	–NH–CH(iPr)–CH_2_–CO–	–NH–CH_2_–CH_2_–C_6_H_5_
3	A-164	*Z*-Arg-	-Leu-	–NH–CH(iPr)–CH_2_–NH–	–PCA
4	A-165	*Z*-Arg-	-Leu-	–NH–CH(iPr)–CH_2_–NH–	–NAA
5	A-174	*Z*-Lys-	-His-	–NH–CH(iPr)–CH_2_–NH–	–Cin
6	A-176	*Z*-Arg-	-His-	–NH–CH(iPr)–CH_2_–NH–	–PCA
7	A-179	*Z*-Arg-	-Arg-	–NH–CH(iPr)–CH_2_–NH–	–PCA
8	A-184	*Z*-Arg-	-Arg-	–NH–CH(iPr)–CH_2_–NH–	–NAA
9	A-191	*Z*-Lys-	-Arg-	–NH–CH(iPr)–CH_2_–NH–	–PCA
10	A-192	*Z*-Har-	-Arg-	–NH–CH(iPr)–CH_2_–NH–	–PCA
11	A-193	*Z*-Har-	-His-	–NH–CH(iPr)–CH_2_–NH–	–PCA
12	A-194	*Z*-Har-	-Arg-	–NH–CH(iPr)–CH_2_–NH–	–NAA
13	A-196	*Z*-Arg-	-Leu-	-Val-	–NH–CH_2_–CH_2_–C_6_H_5_
14	A-197	*Z*-Arg-	-Leu-	–NH–CH(iPr)–CH_2_–NH–	
15	A-198	Arg-	-Leu-	–NH–CH(iPr)–CH_2_–NH–	–CO–CH_2_–CH_2_–CH_2_–C_6_H_5_
16	A-209	*Z*-Arg-	-Leu-	–NH–CH(iPr)–CH_2_–NH–	–CO–CH_2_–CH=CH_2_
17	A-210	*Z*(*o*-CH_3_)-Arg-	-Leu-	–NH–CH(iPr)–CH_2_–NH–	–Cin
18	A-213	*Z*-Arg-	-Leu-	–NH–CH(CH_2_–CH_3_)–CH_2_–NH–	–Cin
19	A-219	*Z*-Arg-	-His-	-Val-	–NH–CH_2_–CH_2_–C_6_H_5_
20	A-220	*Z*-Lys-	-His-	-Val-	–NH–CH_2_–CH_2_–C_6_H_5_
21	A-221	*Z*-Arg-	-Arg-	-Val-	–NH–CH_2_–CH_2_–C_6_H_5_

a P1–P4 are positions of modification. Cin—*trans*-cinnamyl moiety, PCA—α-phenylcinnamyl moiety, NAA—1-naphthylacetic acid moiety.

### Stability in Water and Presence in Human Plasma

Blood was drawn from healthy volunteers, and lithium/heparin holding tubes were used to remove the clot by centrifugation. The plasma was aliquoted in sterile conditions and incubated with the desired compounds for 24 h at 37 °C. The samples were prepared under sterile conditions following the previously described protocol.^27^ The samples were collected at six time points (0, 1, 2, 3, 6, and 24 h) for determining stability in water solutions, and time points of 0, 10, 20, 30, 40, 50, and 60 min, as well as of 2, 3, 6, and 24 h, for determining stability in human plasma. The analysis was performed by HPLC using a Kromasil C8 column (5 μm, 90 Å, 1.0 mm × 250 mm) monitored by a PDA or ELDS-LT II detector. For quantification, peak areas were compared to a calibration curve calculated by Shimadzu LCsolution Software (Kyoto, Japan). Graphs were prepared in GraphPad Prism (Boston, USA).

### Antimicrobial Assay

*S. aureus* (ATCC 29213),*S. pyogenes* (ATCC 19615),*Streptococcus agalactiae* (ATCC 27956),*Escherichia coli* (ATCC 25922),*Proteus vulgaris* (ATCC 6896),*K. pneumoniae* (ATCC 700603) and*P. aeruginosa* (ATCC 10145) reference strains, as well as*Staphylococcus cohnii*, *Enterococcus faecalis*, *Staphylococcus schleiferi*, *Staphylococcus intermedius* and *Streptococcus mutans* clinical isolates, were used to test the antibacterial activity of the peptidomimetics. Bacteria were aerobically cultured for 18 h at 37 °C in plates with sterilized brain heart infusion (BHI) broth (Sigma-Aldrich, USA) media mixed with bacteriological agar (Sigma-Aldrich, USA) using the streak plate method. After 18 h, BHI broth was inoculated to 0.5 on the McFarland scale and diluted to approximately 10^5^ CFU/mL. To achieve a final stock concentration of 1024 μg/mL, the peptidomimetics were dissolved in sterile water, and 5–10% of absolute ethanol or dimethyl sulfoxide (DMSO) was added. The antibacterial activities were determined by using the standard broth microdilution method of the National Committee for Clinical Laboratory Standards. The process involved adding serially diluted peptidomimetics to consecutive wells of a 96-well microtiter plate (Brand, Germany) holding media with microorganisms. The inoculated plates treated with peptides were then placed in an incubator at 37 °C for 18 h. The MIC value was determined as the lowest concentration of peptidomimetic at which there was no obvious growth (equal or more than 90% of the untreated sample). To determine minimum bactericidal concentration (MBC) values, 10 μL of medium from each well with no visible growth was spread on BHI-agar plates and incubated at 37 °C for 18 h. The lowest concentration yielding growth in no more than 3 colonies, corresponding to at least a 99.9% kill rate, was taken as MBC. Each biological replicate included control wells with the addition of water, DMSO, and ethanol as respective solvent controls. All experiments were conducted with consistent adherence to growth and sterility controls and were performed at least in technical triplicate with three biological repeats. MICs were converted to a color scale and displayed by using GraphPad Prism 8 (Boston, USA).

### Cell Culture Conditions

In our study, we used two distinct cell types: immortalized human HaCaT keratinocytes (DKFZ, Heidelberg, Germany) and a human dermal fibroblast cell line (46BR.1N). Both HaCaT and 46BR.1N cells were cultured in Dulbecco’s modified Eagle’s medium (DMEM) (Sigma-Aldrich, USA), containing 4500 μg/mL glucose, 584 μg/mL l-glutamine, sodium pyruvate, and sodium bicarbonate, supplemented with 10% fetal bovine serum (FBS), 100 units/mL penicillin, and 100 μg/mL streptomycin (Sigma-Aldrich, USA). Cultures were maintained in a humidified atmosphere with 5% CO_2_ at 37 °C in 25 cm^2^ culture flasks with the medium replenished every 2 days. The condition of the cells was monitored using optical microscopy.

### Cytotoxicity Test

Cell death in immortalized human keratinocytes (HaCaT) and fibroblasts (46BR.1N) was measured by quantifying the lactate dehydrogenase (LDH) activity using a commercial kit (Roche, Switzerland). Cells were seeded in 96-well plates at a density of 5000 cells per well in DMEM high glucose medium supplemented with 10% FBS. After 24 h of incubation, the medium was replaced with serum-free medium containing various concentrations of peptidomimetics (50, 100, and 150 μg/mL). After 48 h, supernatants were collected for LDH analysis. Plate readings were taken spectrophotometrically using a 450 nm wavelength. Cell death measurements were normalized to an untreated control (0%), with 1% Triton X-100 detergent used as a positive control to induce maximum LDH release, indicating maximal cytotoxicity. Statistical significance was determined with a Mann–Whitney *U* test with a statistical significance *p* ≤ 0.05. Analysis was performed with Statistica software (Krakow, Poland) and graphs were prepared in GraphPad Prism (Boston, USA).

### Cell Proliferation Assay

Cells were seeded into 96-well plates at a density of 5000 cells per well in a medium supplemented with 10% fetal bovine serum (FBS). After 24 h of incubation, the medium was replaced with serum-free HG-DMEM containing appropriate concentrations of peptidomimetics. All solutions used in the experiments were prepared with double-distilled water or with the addition of ethanol under sterile conditions to ensure compatibility with the conditions of the bacteriological assay. For 72 h of incubation, half of the medium was replaced with fresh medium after 48 h, and cells were stimulated with peptides for a second time. The XTT cell proliferation assay was then performed according to the manufacturer’s instructions (Roche, Switzerland). Cells were treated with the peptides for either 48 or 72 h, followed by the addition of XTT reagent, and the plates were then incubated at 37 °C in a 5% CO_2_ environment for 4 h. Plate readings were taken using a standard plate reader at a wavelength of 490 nm (OD = 490 nm). Cell proliferation levels were normalized to the untreated control (100%). Statistical significance was determined with a Mann–Whitney *U* test with statistical significance *p* ≤ 0.05. Analysis was performed with Statistica software, and graphs were prepared in GraphPad Prism.

### ADMET Prediction

Absorption, distribution, metabolism, excretion, and toxicity (ADMET) properties of synthesized compounds were assessed by utilizing advanced online servers: SwissADME^28^ and pkCSM.^29^

### Exploratory Data Analysis

Exploratory data analyses (i.e., principal component analysis (PCA) and two-way hierarchical cluster analysis (2D-HCA)) were performed using R software (v4.0.3) with the following packages: factoextra,^30^ caret,^31^ and ggplot2.^32^ The additional descriptors were calculated using Dragon-7 software,^33^ while more complex electronic descriptors associated with the 3D conformation of the molecule were computed using the MOPAC2016 program.^34^ Prior to these calculations, the geometries of all peptidomimetic derivatives were preoptimized using the PM7 semiempirical Hamiltonian.^35^ This method was chosen for its balance between accuracy and computational efficiency, ensuring reliable results with minimal computational cost.^36^ A detailed list of all calculated molecular descriptors can be found in Table S1.

## Results

### Design and Synthesis

Based on our previous studies^20–23^ we designed and synthesized a series of twenty-one compounds based on the *N*-terminal fragment of human cystatin C (RLVG). The scheme of conversion from Boc-Aa-OH to *N* ^2^-Boc-1,2-diamines, which is a core of these compounds (Figure 1, P3) is represented in Scheme 1. This method was used to synthesize 17 compounds (A-20, A-134, A-164, A-165, A-174, A-176, A-179, A-184, A-191, A-192, A-193, A-194, A-197, A-198, A-209, A-210, and A-213). For elongation from lysine to homoarginine (described as Har in Table 1) in A-134, A-192, A-193, and A-194, we used 3,5-dimethyl-1-pyrazolylformaminidium nitrate, which was then placed in position P1 (Figure 1). For preparation of Boc-βLeu-OH an Arndt–Eistert synthesis was used, as shown in Scheme 2, and this moiety was then placed in position P3 in analogue A-152. In some cases (A-152, A-196, A-219, A-220, and A-221) phenethylamine in position P4 was used to mimic the structure of a cinnamic acid residue. To obtain more sterically hindered analogues in position P4 α-phenylcinnamic acid (named PCA in Table 1) and 1-naphthylacetic acid (named NAA in Table 1) were used (for A-164, A-165, A-176, A-179, A-184, A-191, A-192, A-193, and A-194). To enhance the potential solubility of compounds at position P2, basic residues such as arginine and histidine were incorporated. All peptidomimetics were purified using a HPLC system to at least 95% of purity and characterized by mass spectrometry (Table S2).

**Figure 1:**
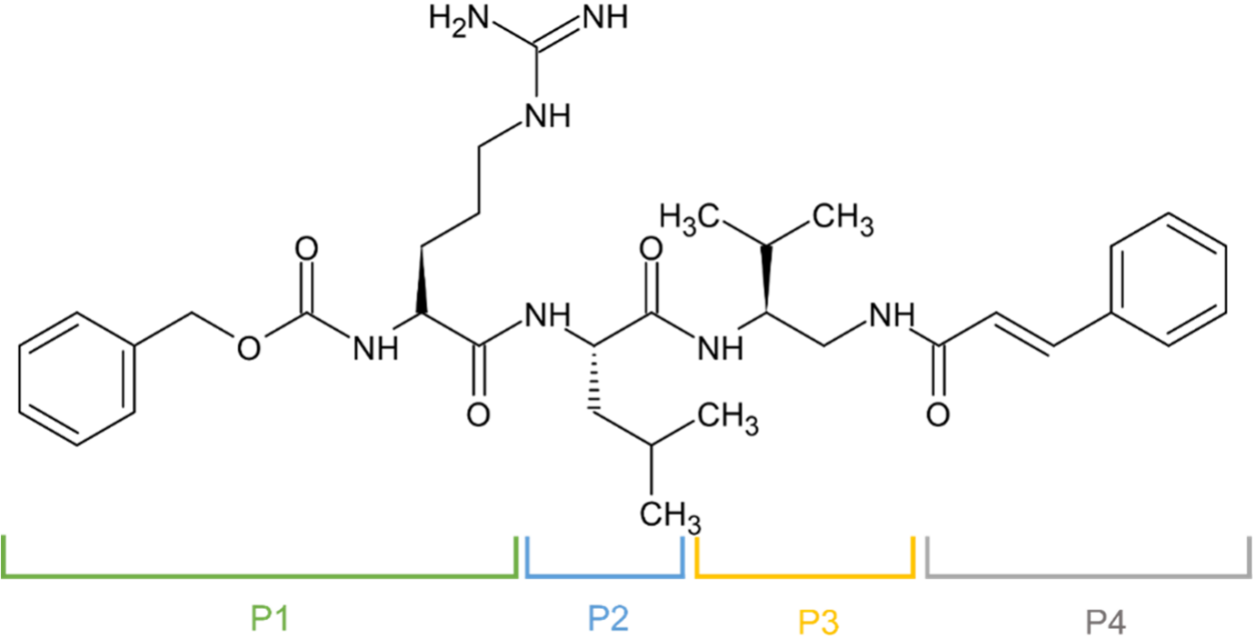
Structure of A-20, the molecule upon which all analogues were based, showing positions of modifications.

### Hydrophobicity Parameters

Hydrophobicity of antibiotics influences their ability to attach to and penetrate bacterial cell membranes, directly affecting their antimicrobial potency. In addition, hydrophobicity affects the compound’s solubility, tissue distribution, and interaction with molecular targets, playing a pivotal role in determining pharmacokinetics and the ability to localize at infection sites.

Reverse-phase chromatography was employed to assess the hydrophobicity characteristics of the peptidomimetics. Hydrophobicity was quantified by determining the acetonitrile content in the mobile phase at the retention time of a peptidyl derivative (Table S2). The least hydrophobic compound (40% acetonitrile) was A-198, which aligns with the calculations of hydrophobicity by SwissADME and pkCSM (Table 2). Next in the hydrophobicity sequence were A-197, A-220, A-219, A-174, and A-221, eluting with 42.5–43.9% of acetonitrile. Then there were followed by A-191, A-194, A-184, A-192, A-193, A-209, and A-179, with the acetonitrile percentage of 44.8–49.8. The compounds A-176, A-20, A-152, A-134, and A-210 needed over 50% of acetonitrile to be eluted from the column. The compounds with the poorest solubility and most hydrophobic properties were A-196, A-164, and A-165, all of which needed more than 60% of acetonitrile to be eluted from the column.

**Table 2:** ADMET Parameters Calculated by Algorithms SwissADME and pkCSMY for All 22 Compounds

descriptor	A-20	A-134	A-152	A-164	A-165	A-174	A-176	A-179	A-184	A-191	A-192	A-193
Adsorption												
lipophilicity												
Log *P*_o/w_ (iLOGP)	2.87	4.52	3.19	3.99	4.49	3.74	–2.61	2.84	1.53	3.85	3.02	3.30
Log *P*_o/w_ (XLOGP3)	4.23	4.58	4.03	5.86	4.99	3.26	4.38	3.72	2.85	4.31	4.08	4.66
Log *P*_o/w_ (WLOGP)	2.79	3.18	2.81	4.31	3.58	2.57	3.23	2.53	1.79	3.40	2.92	3.62
Log *P*_o/w_ (MLOGP)	1.85	2.03	1.92	2.70	2.28	0.54	1.06	1.62	1.20	1.95	1.79	1.23
Log *P*_o/w_ (SILICOS-IT)	4.12	4.54	4.30	5.47	4.94	4.50	4.64	3.75	3.23	4.82	4.18	5.18
consensus Log *P*_o/w_	3.17	3.77	3.25	4.47	4.06	2.92	2.14	2.89	2.12	3.66	3.20	3.60
water solubility												
Log *S* (ESOL)	–5.06	–5.29	–4.88	–6.55	–5.82	–4.57	–5.83	–5.26	–4.53	–5.53	–5.50	–6.02
solubility [μg/mL]	5.59	3.31	8.48	0.20	1.02	17.2	1.10	4.16	21.4	2.14	2.42	40.71
class	moderately soluble	moderately soluble	moderately soluble	poorly soluble	moderately soluble	moderately soluble	poorly soluble	moderately soluble	moderately soluble	moderately soluble	moderately soluble	poorly soluble
Log *S* (Ali)	–7.88	–8.24	–7.67	–9.57	–8.67	–6.72	–8.64	–8.65	–7.75	–8.51	–9.02	–8.93
solubility [μg/mL]	8.40 × 10^–3^	3.72 × 10^–3^	1.36 × 10^–2^	1.91 × 10^–4^	1.45 × 10^–3^	1.20 × 10^–1^	1.70 × 10^–3^	1.69 × 10^–3^	1.28 × 10^–2^	2.26 × 10^–3^	7.29 × 10^–4^	8.87 × 10^–4^
class	poorly soluble	poorly soluble	poorly soluble	poorly soluble	poorly soluble	poorly soluble	poorly soluble	poorly soluble	poorly soluble	poorly soluble	poorly soluble	poorly soluble
Log *S* (SILICOS-IT)	–8.04	–8.79	–9.12	–10.81	–10.34	–9.20	–10.64	–10.52	–10.05	–10.82	–10.90	–11.69
solubility [μg/mL]	2.52 × 10^–3^	1.06 × 10^–3^	4.86 × 10^–4^	1.11 × 10^–5^	3.09 × 10^–5^	4.00 × 10^–4^	1.67 × 10^–5^	2.31 × 10^–5^	6.43 × 10^–5^	1.11 × 10^–5^	9.75 × 10^–6^	1.55 × 10^–6^
cass	poorly soluble	poorly soluble	poorly soluble	insoluble	insoluble	poorly soluble	insoluble	insoluble	insoluble	insoluble	insoluble	insoluble
pharmacokinetics												
GI absorption	low	low	low	low	low	low	low	low	low	low	low	low
P-glycoprotein substrate	yes	yes	yes	yes	yes	yes	yes	yes	yes	yes	yes	yes
Log *K*_p_ (skin permeation) [cm/s]	–7.18	–7.07	–7.33	–6.48	–6.87	–7.84	–7.68	–8.26	–8.65	–7.67	–8.09	–7.57
Distribution												
BBB permeant	no	no	no	no	no	no	no	no	no	no	no	no
VDss (human) [log L/kg]	–0.226	–0.198	–0.267	–0.436	0.106	–0.237	–0.116	–0.244	–0.246	–0.251	–0.205	–0.089
Metabolism												
CYP2D6 substrate	no	no	no	no	no	no	no	no	no	no	no	no
CYP3A4 substrate	yes	yes	yes	yes	yes	yes	yes	yes	yes	yes	yes	yes
CYP1A2 inhibitor	no	no	no	no	no	no	no	no	no	no	no	no
CYP2C19 inhibitor	no	no	no	no	no	no	no	no	no	no	no	no
CYP2C9 inhibitor	yes	yes	yes	yes	yes	no	no	yes	no	yes	yes	no
CYP2D6 inhibitor	no	no	no	no	no	no	no	no	no	no	no	no
CYP3A4 inhibitor	no	no	no	no	no	yes	no	no	no	no	no	no
Excretion												
total clearance [log mL/min/kg]	0.094	0.116	0.193	–0.36	0.293	0.577	0.204	–0.056	0.157	0.127	–0.065	0.206
renal OCT2 substrate	no	no	no	no	no	no	no	no	no	no	no	yes
Toxicity												
AMES toxicity	no	no	no	no	no	no	no	no	no	no	no	no
hepatotoxicity	yes	yes	yes	yes	yes	yes	yes	yes	yes	yes	yes	yes
skin sensitization	no	no	no	no	no	no	no	no	no	no	no	no

descriptor	A-194	A-196	A-197	A-198	A-209	A-210	A-213	A-219	A-220	A-221
Adsorption										
lipophilicity										
Log *P*_o/w_ (iLOGP)	2.86	2.72	2.77	2.67	3.54	4.04	3.95	2.08	2.9	1.74
Log *P*_o/w_ (XLOGP3)	3.27	4.10	1.89	1.88	2.89	4.20	3.79	2.54	3.13	1.96
Log *P*_o/w_ (WLOGP)	2.26	2.42	1.02	1.00	1.76	2.79	2.54	1.34	2.21	0.64
Log *P*_o/w_ (MLOGP)	1.47	1.74	0.83	0.99	1.13	1.55	1.67	0.09	0.43	0.66
Log *P*_o/w_ (SILICOS-IT)	3.23	3.87	1.83	1.69	3.17	4.24	3.86	3.19	4.25	2.16
consensus Log *P*_o/w_	2.62	2.97	1.67	1.84	2.50	3.36	3.26	1.85	2.59	1.43
water solubility										
Log *S* (ESOL)	–4.79	–4.90	–2.97	–2.95	–3.74	–5.15	–4.70	–4.14	–4.42	–3.61
solubility [μg/mL]	1.16	7.78	543	564	104	4.72	12.5	46.9	23.8	163
class	moderately soluble	moderately soluble	soluble	soluble	soluble	moderately soluble	moderately soluble	moderately soluble	moderately soluble	soluble
Log *S* (Ali)	–8.81	–7.74	–5.39	–5.18	6.49	–8.04	–7.42	–6.73	–6.59	–6.82
solubility [μg/mL]	4.71 × 10^–3^	1.12 × 10^–2^	2.08	3.31	1.86 × 10^–1^	6.05 × 10^–3^	2.35 × 10^–2^	1.21 × 10^–1^	1.61 × 10^–1^	1.00 × 10^–1^
class	poorly soluble	poorly soluble	moderately soluble	moderately soluble	poorly soluble	poorly soluble	poorly soluble	poorly soluble	poorly soluble	poorly soluble
Log *S* (SILICOS-IT)	–10.05	–8.73	–5.60	–6.27	–6.73	–8.49	–8.39	–9.23	–9.53	–8.45
solubility [μg/mL]	6.43 × 10^–5^	1.15 × 10^–3^	1.26	2.72 × 10^–1^	1.07 × 10^–1^	2.17 × 10^–3^	2.53 × 10^–3^	3.80 × 10^–4^	1.83 × 10^–4^	2.39 × 10^–3^
cass	insoluble	poorly soluble	moderately soluble	poorly soluble	poorly soluble	poorly soluble	poorly soluble	poorly soluble	poorly solule	poorly solubly
pharmacokinetics										
GI absorption	low	low	low	low	low	low	low	low	low	low
P-glycoprotein substrate	yes	yes	yes	yes	yes	yes	yes	yes	yes	yes
Log *K*_p_ (skin permeation) [cm/s]	–8.35	–7.19	–8.04	–8.04	–7.75	–7.38	–7.40	–8.45	–7.86	–8.98
Distribution										
BBB permeant	no	no	no	no	no	no	no	no	no	no
VDss (human) [log L/kg]	–0.243	–0.623	–0.695	0.482	–0.757	–0.222	–0.205	–0.309	–0.473	–0.532
Metabolism										
CYP2D6 substrate	no	no	no	no	no	no	no	no	no	no
CYP3A4 substrate	yes	yes	yes	yes	yes	yes	yes	yes	yes	yes
CYP1A2 inhibitor	no	no	no	no	no	no	no	no	no	no
CYP2C19 inhibitor	no	no	no	no	no	no	no	no	no	no
CYP2C9 inhibitor	yes	yes	no	no	yes	yes	yes	no	no	no
CYP2D6 inhibitor	no	no	no	no	no	no	no	no	no	no
CYP3A4 inhibitor	no	no	no	no	no	no	no	no	yes	no
Excretion										
total clearance [log mL/min/kg]	0.023	0.006	–0.018	0.458	0.106	–0.024	0.265	0.212	0.422	–0.346
renal OCT2 substrate	no	no	no	no	no	no	no	no	no	no
Toxicity										
AMES toxicity	no	no	no	no	no	no	no	no	no	no
hepatotoxicity	yes	yes	no	no	yes	yes	yes	yes	yes	yes
skin sensitization	no	no	no	no	no	no	no	no	no	no

### Stability in Water and Presence in Human Plasma

Stability in water and human plasma is crucial for peptides and peptidomimetics, as it influences their bioavailability, half-life, and therapeutic efficacy. High stability ensures that these molecules resist enzymatic degradation and hydrolysis, enabling sustained biological activity and improved pharmacokinetic profiles. For stability determination, peptidomimetics were incubated both in water and in human plasma. The samples were collected at 0, 1, 2, 3, 6, and 24 h. The peptidomimetics were stable in water for at least 24 h (Figure 2—pink), and almost all peptidomimetics were stable in human plasma for at least 24 h (Figure 2—green). Only A-196, A-219, A-220, and A-221 showed slight disappearance from plasma, yet still being 85–90% present. Exemplary chromatograms can be found in Figure S1.

**Figure 2:**
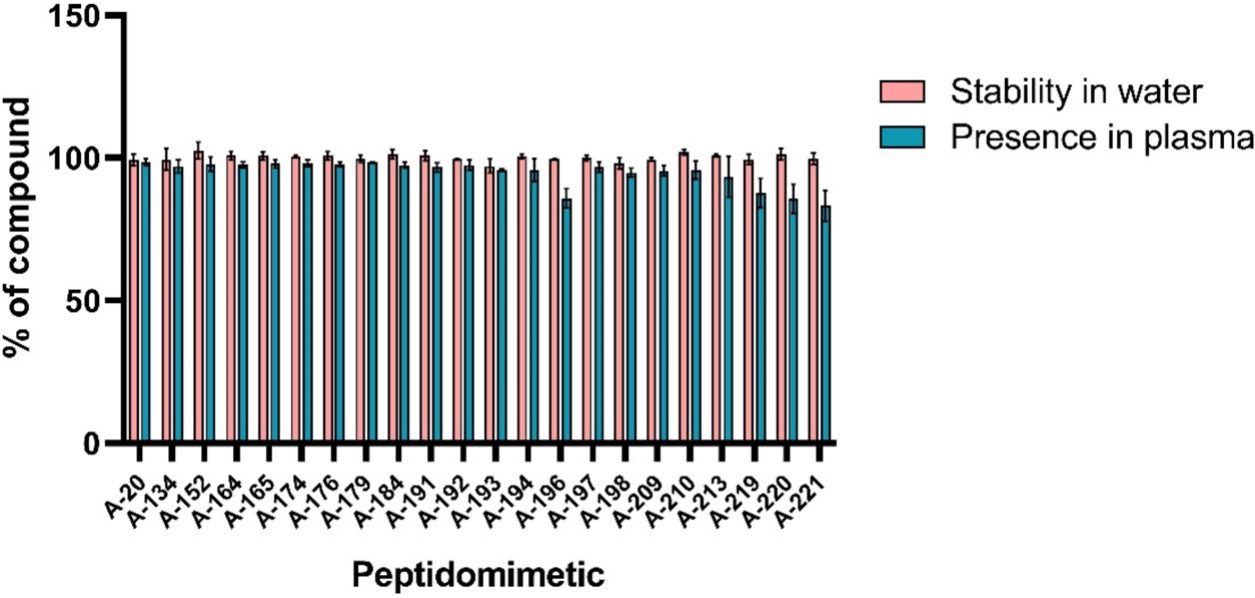
Stability of peptidomimetic in water after 24 h of incubation and detection of the quantity presence of peptidomimetic in human plasma during 24 h of incubation.

### Antimicrobial Activity

Figure 3 shows a heatmap of some MIC peptidomimetic values across different bacterial strains; a full list of MIC and MBC values is provided in Supporting Information (Table S3).

**Figure 3:**
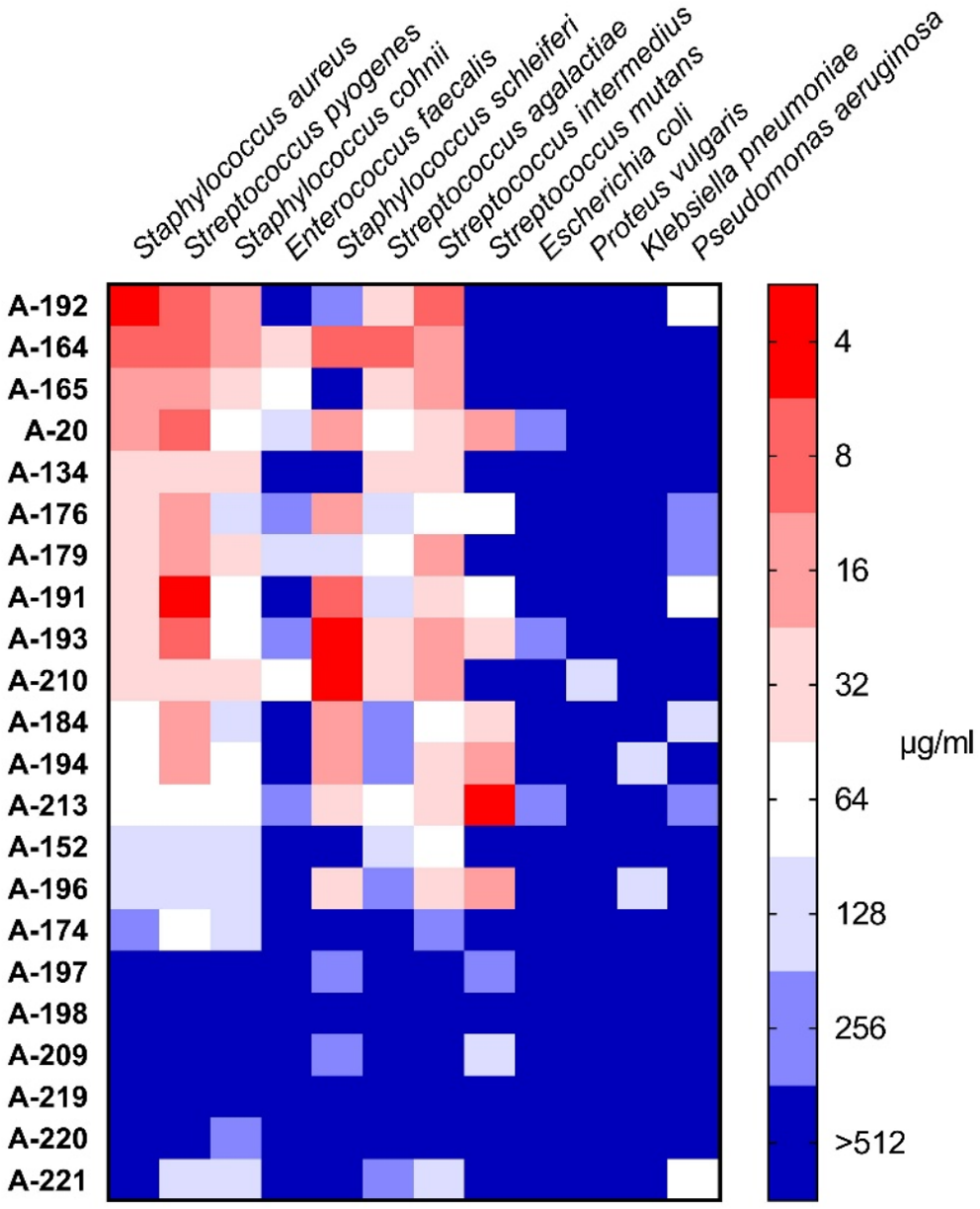
Heatmap of minimum inhibitory concentration (MIC) values for peptidomimetics across different bacterial strains. The color scale indicates the MIC values in μg/mL, with red representing lower MIC values (more potent inhibition) and blue representing higher MIC values (less potent inhibition).

From the heatmap it is easily seen that compounds A-192 and A-164 display the most promising antibacterial properties, making them strong candidates for further investigation. A-192 was most potent against *S. aureus* strain, but was also potent against *S. pyogenes*, *S. cohnii* and *S. intermedius*. A-164 was active for both *S. aureus* and *S. pyogenes*, *S. schleiferi*, and *S. agalactiae*. Next active compound was A-191, the activity of which was mostly on *S. pyogenes* and *S. schleiferi*. A-210 seemed to be active against *S. schleiferi*, while A-213 was active against *S. mutans*. In contrast, the activity of the tested compounds against Gram-negative bacteria, including *E. coli*, *P. vulgaris*, *K. pneumoniae*, and *P. aeruginosa*, was largely absent, with MIC values exceeding 512 μg/mL in most cases. Some compounds, such as A-220 and A-221, showed little to no activity across all tested bacterial strains, indicating their limited therapeutic potential.

### ADMET Studies

Computed absorption, distribution, metabolism, excretion, and toxicity (ADMET) predictions were calculated for potential drugs to evaluate their pharmacokinetic and toxicological properties before experimental testing. These computational models help identify candidates with favorable drug-like characteristics, reducing costs and time in drug development by filtering out compounds likely to exhibit poor bioavailability or adverse effects. A web tool called SwissADME was used for evaluating the pharmacokinetic properties of small molecules. The second tool used was pkCSM, which has a wider spectrum of parameters and completes the missing data. The ADMET properties of all compounds are shown in Table 2.

### Cytotoxicity Assay of Peptidomimetics

An LDH assay was performed to quantify cytotoxicity induced by potential drug candidates, as increased LDH levels in the extracellular environment indicate cell damage or death.

The cytotoxicity of the tested compounds was assessed within 48 h of incubation with cells (keratinocytes and fibroblasts) and compared to the 1% Triton X-100 control. The results are shown in Figure 4. The cytotoxicity of A-20 was previously reported.^22^ Compounds A-174 and A-197 showed no cytotoxicity. Compound A-191 showed a high cytotoxic effect only to fibroblasts at the highest tested concentration. At lower concentrations, this effect faded away. Compound A-196 displayed a slight cytotoxic effect (∼20%) for keratinocytes at a concentration of 150 μg/mL. Compound A-198 showed mild cell toxicity (∼20%) specifically toward fibroblasts. Compound A-213 exerted cytotoxicity (∼10%) at a concentration of 150 μg/mL. A-193 seemed to be cytotoxic only at a concentration of 150 μg/mL, and it was more cytotoxic to keratinocytes than to fibroblasts at this concentration. Both A-219 and A-221 exhibited slight cytotoxicity toward fibroblasts across all concentrations, while demonstrating no adverse effects on keratinocytes. A-194, A-209, and A-220 displayed mild toxicity (∼10–15%) toward both cell lines. However, A-194 exhibited a notable increase to over 40–50% toxicity at a concentration of 150 μg/mL. A-134 and A-152 exerted cytotoxicity at every studied concentration, although not exceeding 50%. A-176 had a negative effect on both keratinocytes (over 50%) and fibroblasts, and this effect became stronger with increasing concentration. A-210 seemed to have no negative effect at 50 μg/mL concentration, but a cytotoxic effect, which was stronger toward keratinocytes, appeared at 100 μg/mL. Both A-164 demonstrated cytotoxic effects across all of the tested concentrations. The cytotoxic effect of A-165 was stronger with increasing concentration. A-179 was cytotoxic to keratinocytes at all concentrations; however, this effect was less pronounced in fibroblasts. A-192 seemed to have a more negative effect on keratinocytes than on fibroblasts at all concentrations (∼20% cytotoxicity). A-184 seemed to have a negative effect on both cell lines, and its toxic effect was over 60%.

**Figure 4:**
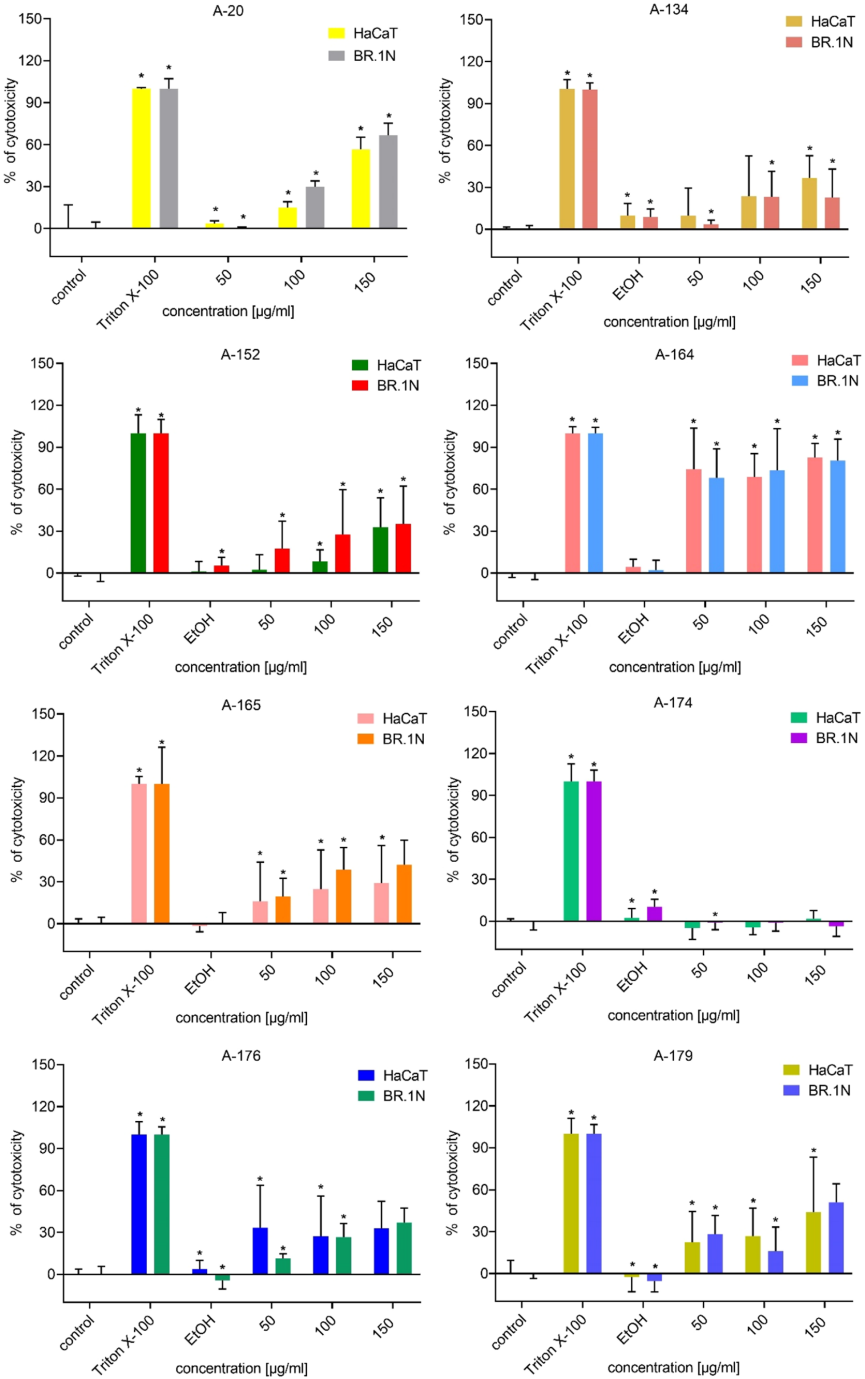

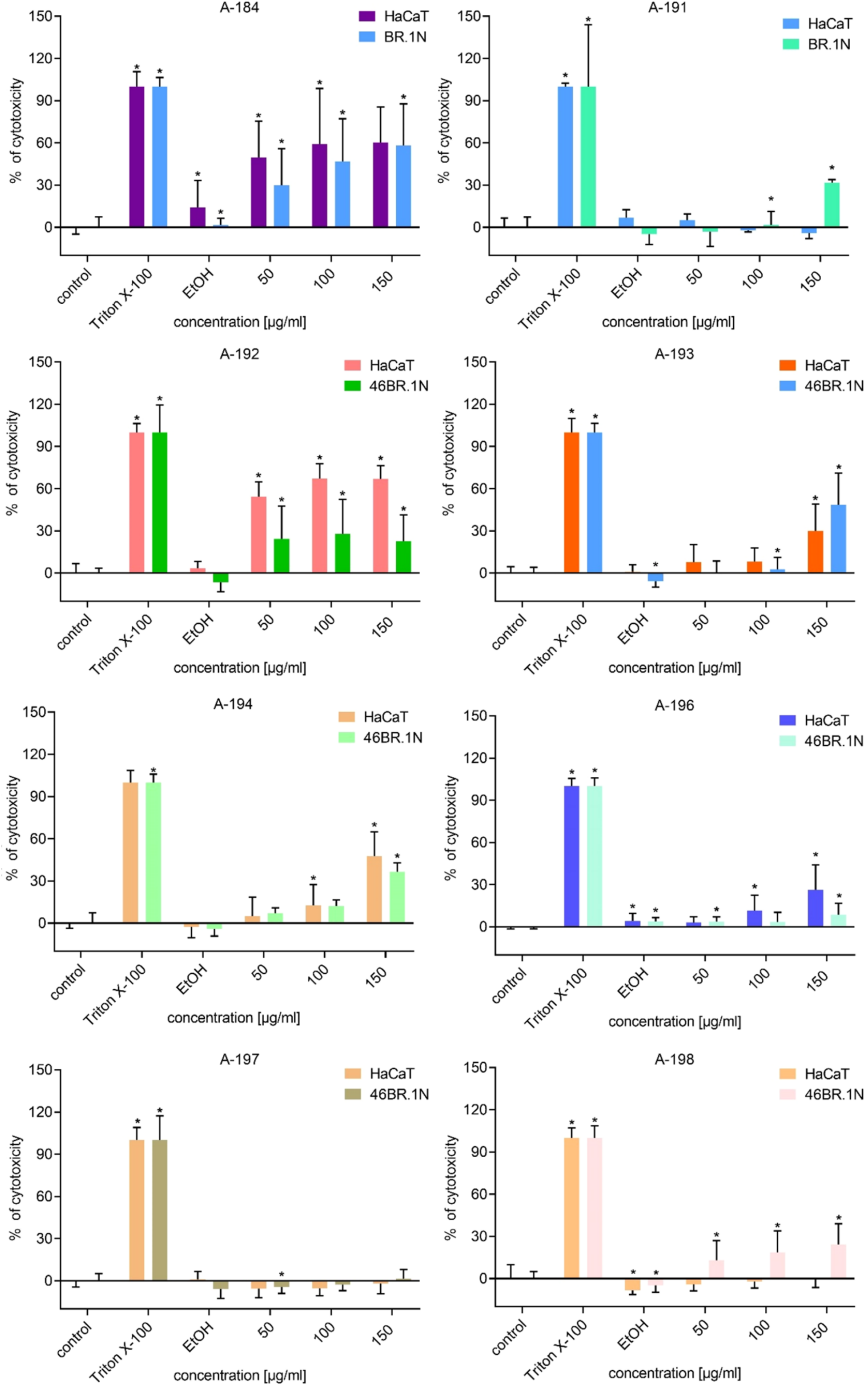

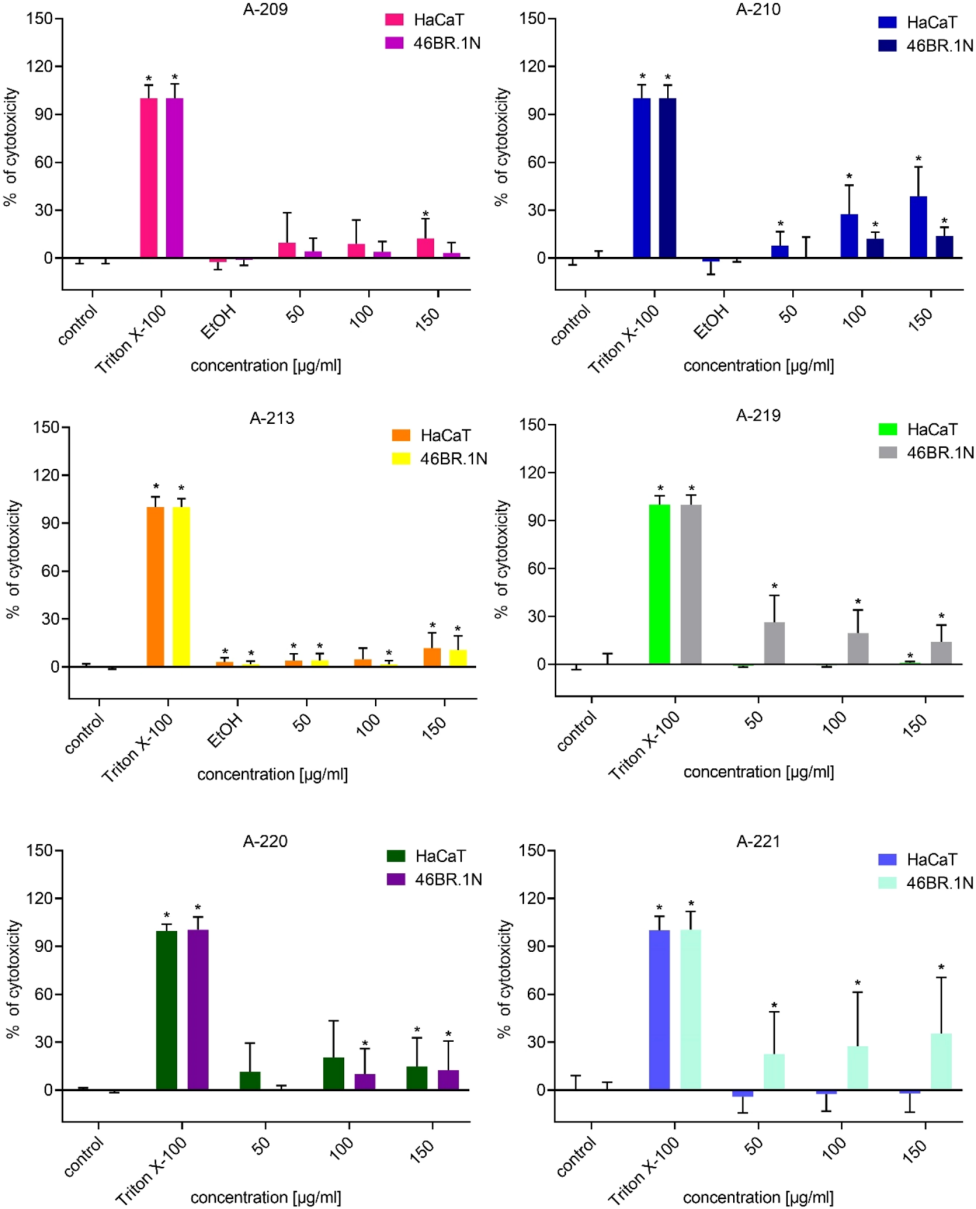
Results of LDH assays on both keratinocytes and fibroblasts in 48 h of incubation. *p* < 0.05 (*U* Mann–Whitney, *n* = 3).

### Cell Proliferation Assay

XTT tests on keratinocytes and fibroblasts were conducted to assess the influence of potential drug candidates on cells proliferation. These cell types are representative of skin tissue, and their response to the compounds provides insights into possible adverse effects, such as the inhibition of proliferation, that could impact skin integrity or wound healing during therapeutic application.

A viability test for A-20 has been previously reported.^22^ Cell proliferation assay was conducted for all compounds for 48 and 72 h of incubation, and the graphs are shown in Figure 5. Compounds exerting a pro-proliferating effect at lower concentrations followed by an antiproliferative effect at higher concentrations were A-134, A-152, A-213, and A-221. A-134 showed a pro-proliferative effect for both keratinocytes and fibroblasts at concentrations of 0.01, 0.1, 1.0, 10, and 25 μg/mL, and this effect was stronger after 72 h of incubation. At a concentration of 50 μg/mL, the proliferation was limited to approximately 75% and at both 100 and 150 μg/mL concentrations, it showed an antiproliferative effect. A-152 showed a pro-proliferative effect at concentrations ranging from 0.01 to 50 μg/mL, though only after 72 h of incubation. After 48 h, it did not exert a pro-proliferative effect. For both cell lines, concentrations of 100 and 150 μg/mL A-152 had a negative impact on cell viability. A-213 showed a pro-proliferative effect at concentrations of 0.01, 0.1, 1.0, and 10 μg/mL and was more prominent for keratinocytes after 72 h of incubation and fibroblasts at 48 h, as well as at 72 h. The concentration of 25 μg/mL was around the control, and after that, the proliferation dropped to around 25% at 150 μg/mL. The viability of cells under the influence of A-221 was the best after 48 h for fibroblasts and after 72 h, it was at the level of control. High concentrations (100–150 μg/mL) seemed to decrease proliferation. A-219 had the best pro-proliferative effect on both cell lines. The concentrations of 0.01 and 0.1 μg/mL were at the level of control; however, at higher concentrations the effect started to be more prominent (around 20% higher than control). Peptides A-174, A-197, A-198, A-209, and A-220 did not exert any negative impact on the viability of the studied cell lines. Peptide A-174 seemed to have a subtle pro-proliferative effect, at all concentrations for both tested cell lines. A-197 had no effect on both cell lines after 48 h, though after 72 h an antiproliferative effect was seen at higher concentrations. A similar effect was observed for A-198, where a small pro-proliferative effect was observed after 48 h of incubation. A-209 exerted neither positive nor negative effects on cells after 48 h incubation, but a mild negative effect was observed in 72 h; however, this effect was constant starting from 25 μg/mL. A-220 exerted a pro-proliferative effect only after 72 h and only for keratinocytes and at concentrations ranging from 0.01 to 50 μg/mL, then began to be cytotoxic. On other cell lines, it had no effect or only a slight antiproliferative effect. Peptidomimetics A-193, A-194, and A-196 had a similar impact on the tested cell lines. For A-193, after 48 h, an antiproliferative effect was observed, and it reached over 50% at a concentration of 150 μg/mL. After 72 h, it had a slight pro-proliferative effect on cells at a concentration of 50 μg/mL, though this diminished at 100–150 μg/mL. The effect was stronger for keratinocytes. A-194 had no negative impact on cell viability at a concentration of 50 μg/mL, however, an antiproliferative effect started at 100–150 μg/mL after 48 and 72 h of incubation. A pro-proliferative effect was seen after 72 h on both keratinocytes and fibroblasts; however, this effect was stronger for keratinocytes. Peptide A-196 appeared to have the same effect on both cell lines. At lower concentrations (0.01–10 μg/mL), it showed a pro-proliferative effect of 5–20%, while at higher concentrations (150 μg/mL), it showed an antiproliferative effect of up to 50%. Peptide A-179 exerted negative impact over 48 h for keratinocytes and fibroblasts. This was observed at all concentrations, and it gradually increased with concentration, reaching about a 50% decrease in cell viability in comparison to the control. The effect was less prominent after 72 h. Peptide A-184 had the same effect. Peptides A-164, A-165, A-176, A-191, A-192 and A-210 showed a negative impact on the proliferation of skin cells. In the case of A-164 the negative impact started at 0.01 μg/mL. Although this impact was seen at every level of concentration, it seemed not to exceed 50%. Only incubation of fibroblasts for 48 h at 50, 100, and 150 μg/mL seemed to have the most negative impact, dropping the viability to a bit over 20%. A-165 had almost the same effect. Over 48 h of incubation seemed to do no harm to cells at concentrations not exceeding 50 μg/mL. A slight addition of A-176 to cell culture caused their proliferation to stop, however it did not exceed 50%, except for fibroblasts incubated for 48 h with concentrations of 100 or 150 μg/mL of this peptide. A-191 seemed to limit proliferation in every concentration. At peptide concentrations of 100 and 150 μg/mL it showed proliferation of 20% or less, relative to the control. A-192 showed the same pattern as A-191 and limited the proliferation to 25% in comparison to the control. A-210 seemed to have little negative impact on both cell lines over 48 h, though after 72 h the impact started to be negative. Only keratinocytes showed a percentage drop in proliferation to less than 50% at 72 h.

**Figure 5:**
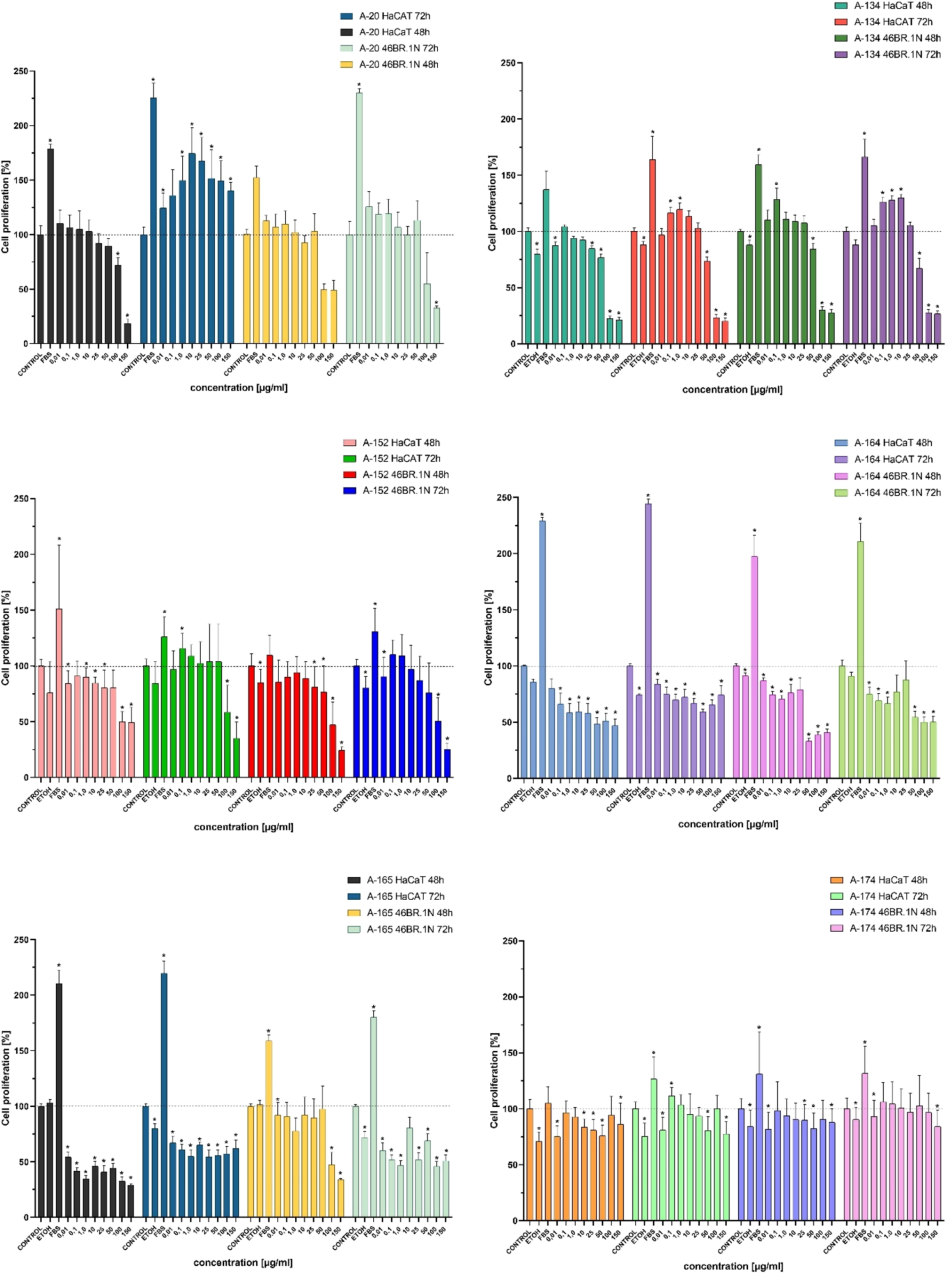

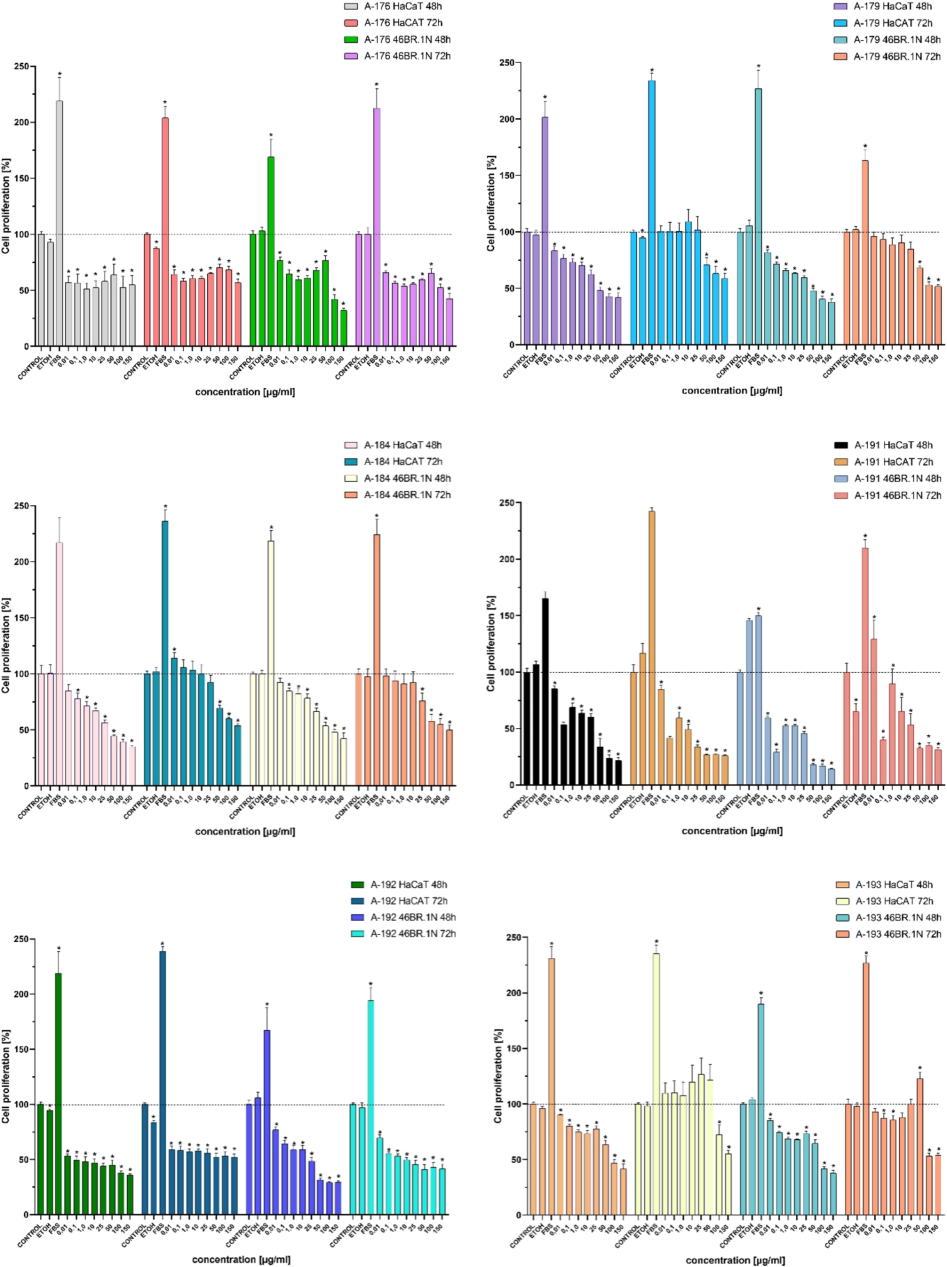

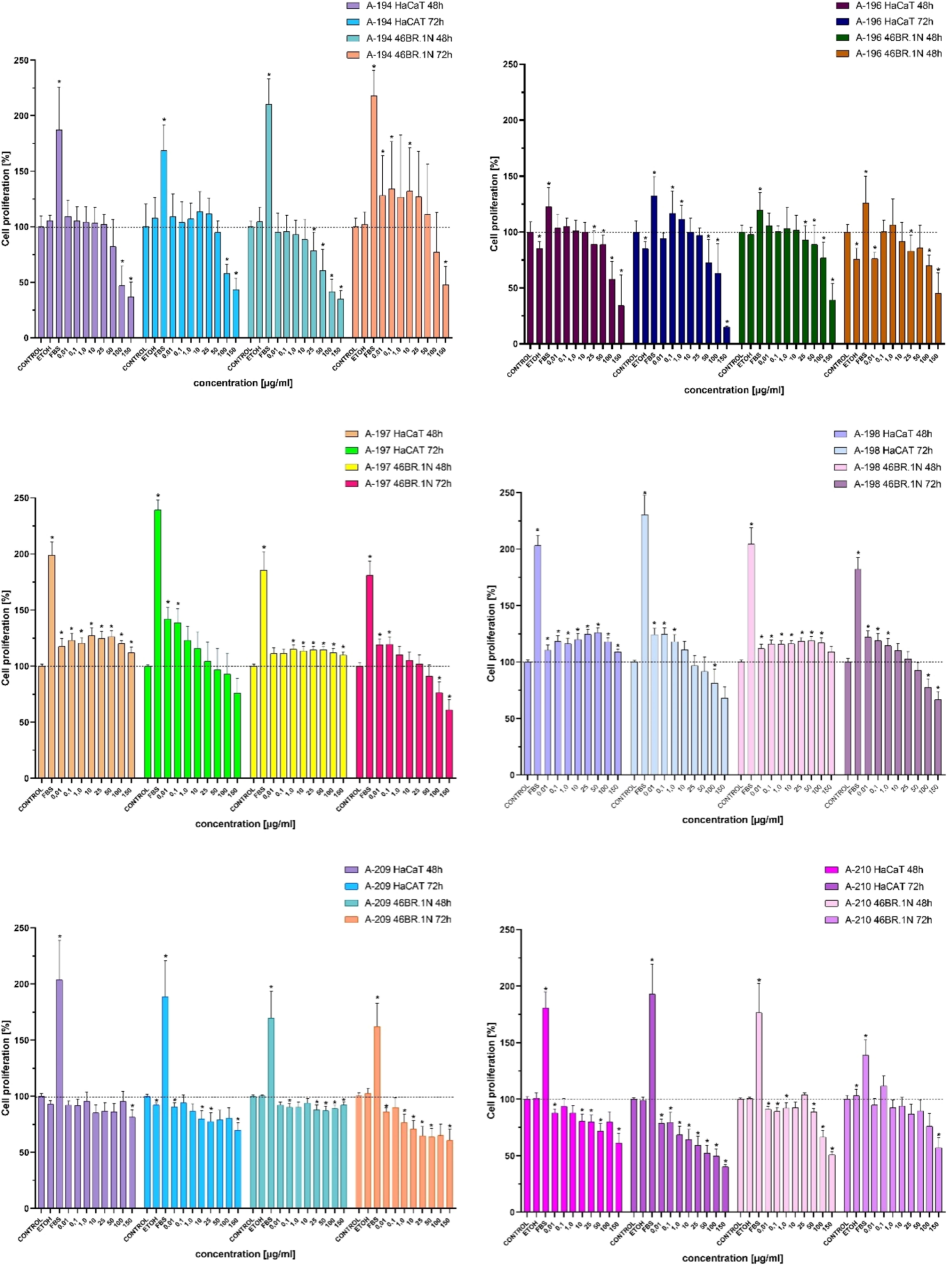

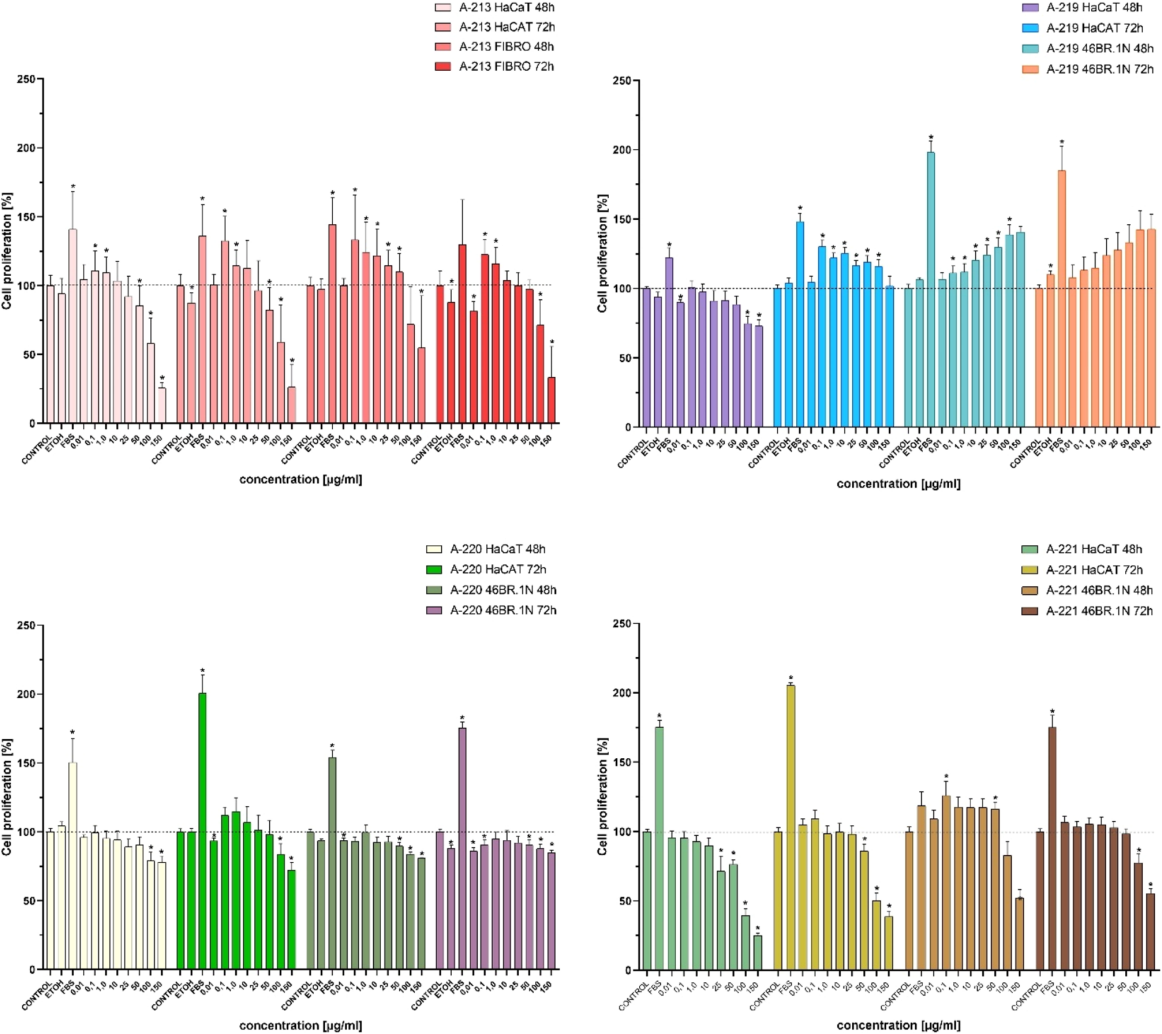
Results of XTT assays on both keratinocytes and fibroblasts in 48 and 72 h incubation. *p* < 0.05 (*U* Mann–Whitney, *n* = 3).

### Exploratory Data Analysis

The initial data set for exploratory data analysis consisted of 169 parameters, including 16 ADME-related parameters and 153 molecular descriptors (Table S1). Analyzing and interpreting such a large and complex set of variables can pose significant challenges, particularly in terms of readability and drawing meaningful conclusions. To address this issue, the first step in the analysis was to apply dimensionality reduction techniques to simplify the data set while retaining the most relevant features. Dimensionality reduction is a process that aims to minimize the number of variables while retaining the most important features that contribute to the overall variability of the data. This can be accomplished through two primary approaches: feature selection and feature extraction. Feature selection focuses on identifying and retaining only the most relevant features by filtering out redundant or less informative features. On the other hand, feature extraction generates new, meaningful features from the original dataset, often combining or transforming existing variables to capture the underlying structure of the data. A common technique that supports both approaches is principal component analysis (PCA), which is widely used to reduce the dimensionality of the data. In this study, PCA was performed iteratively to systematically reduce the number of variables. At each iteration, only those parameters identified as both statistically significant and biologically relevant were retained. Through this iterative process, the initial pool of 169 variables was reduced to a more manageable subset of 15 critical features. These variables included: topological polar surface area (TPSA); solvent accessible surface area (area); synthetic accessibility; molecular volume (volume); first Zagreb index by valence vertex degrees (ZM1 V); molecular weight (MW); Bertz branching index (BBI); Narumi simple topological index (SNar); Dragon branching index (DBI); overall modified Zagreb index of order 0 and 1 by valence vertex degrees (ON0 V, ON1 V); skin permeation (Log *K*_p_); water solubility (Log *S*.ESOL); chemical potential (chem.potential) and the energy difference between highest occupied molecular orbital and lowest unoccupied molecular orbital (GAP). These were then used in the next steps of exploratory data analysis, including PCA for a detailed variance exploration and 2D-HCA.

PCA was conducted to provide a comprehensive understanding of which structural features and/or physicochemical properties of the peptidomimetic derivatives are the most influential in the original dataset and to explore the relationship between: (i) objects (i.e., peptidomimetic derivatives), (ii) variables (i.e., structural and physicochemical properties), and (iii) objects and variables. PCA is a powerful unsupervised machine learning technique that identifies interpretable patterns and trends in data that may elude human observation, while also reducing the complexity inherent in high-dimensional data sets. This is accomplished by identifying orthogonal projections that maximize the variance within the data, termed principal components (PCs). These PCs are derived as linear combinations of the original variables (in this case, molecular descriptors encoding the structure and physicochemical properties of the peptidomimetic derivatives). The aim is for the first principal component (PC1) to explain as much variance in the original data as possible, with subsequent principal components accounting for progressively less variance.

Additionally, to thoroughly explore the relationship between the analyzed peptidomimetic derivatives and their biological activity, this study also conducted a two-way hierarchical cluster analysis (2D-HCA). This approach integrates a dendrogram with a heatmap to illustrate the clustering of compounds based on their properties and the grouping of physicochemical properties. 2D-HCA, acting as a pattern recognition algorithm, relies on distance metrics to quantify the similarity between variables/observations. Herein, Euclidean distance, representing the shortest distance between two points in *n*-dimensional property space, served as the similarity measure, and Ward linkage, employed to minimize variance within clusters, functioned as the agglomeration method, facilitating the evaluation of similarity patterns in the data.

The first two principal components collectively account for 81.30% (68.25 + 13.05%) of the total variance in the data, clearly demonstrating the clustering of peptidomimetic derivatives based on their physicochemical properties and biological activity. While slight variations may arise depending on antibacterial activity (here: against *S. aureus* and *S. pyogenes*) or the cytotoxic activity (here: keratinocytes and fibroblasts), the overarching conclusion remains consistent. Figure 6a,b illustrates that inactive peptidomimetic derivatives are situated on the left side of the biplot. Moving along the first principal component (*X*-axis), compounds progress from inactivity to moderate activity and finally to high activity against Gram-positive bacteria (*S. aureus* and *S. pyogenes*).

**Figure 6:**
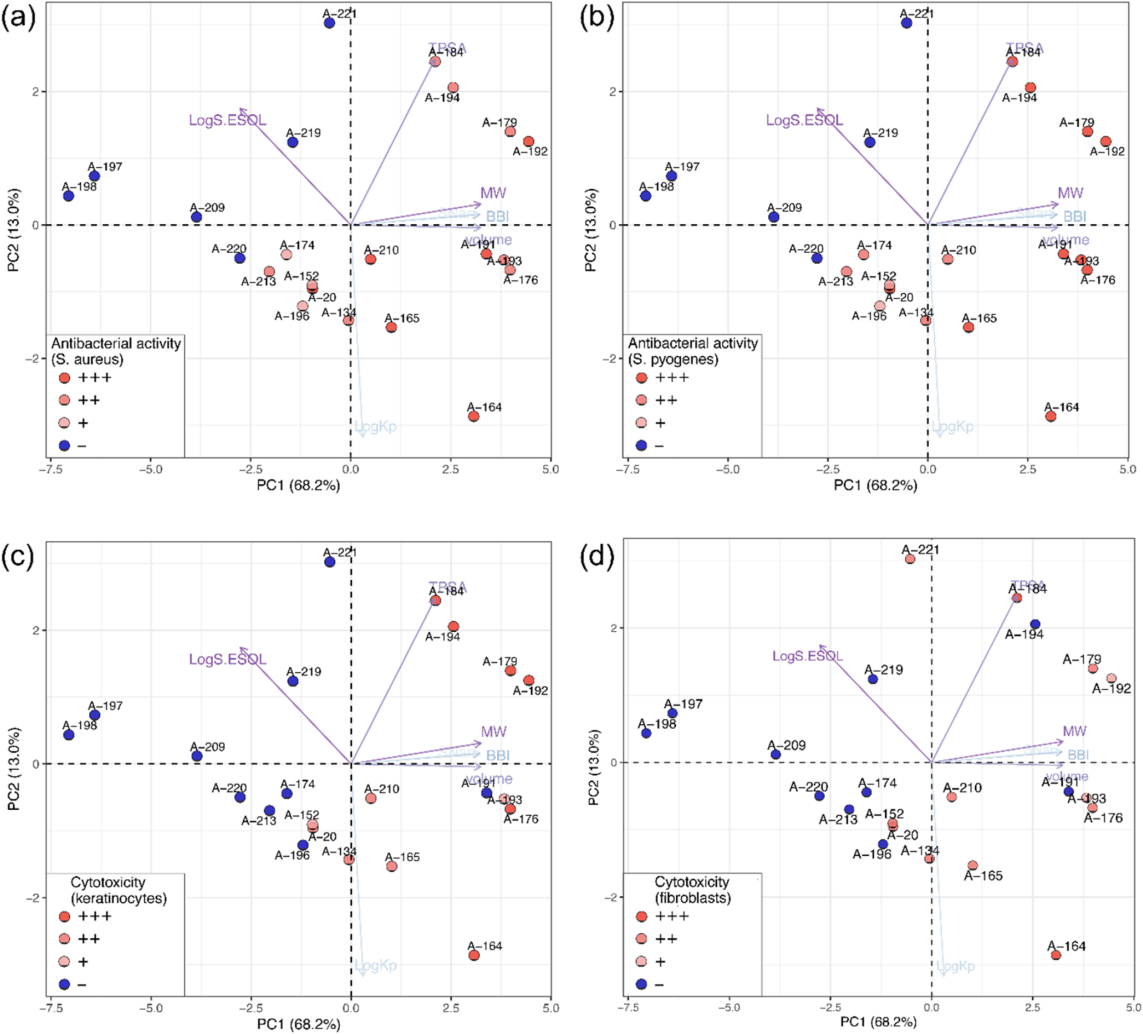
PCA biplot: (a) antibacterial activity against *S. aureus*; (b) antibacterial activity against *S. pyogenes*; (c) cytotoxicity assay in human keratinocyte cell line; (d) cytotoxicity on human fibroblast cells. The PCA biplot simultaneously displays the principal component (PC) values of the peptidomimetic derivatives (represented as data points) and the loadings of the explanatory variables (represented as vectors). The length and transparency of the vector represent the variance explained by the variable and its contribution to the PCs, respectively. Variables with the greatest influence on a particular PC are shown as the longest vectors with the most intense coloring. The angles between the vectors indicate correlations: a small angle indicates a strong positive correlation (e.g., MW, BBI, SNar, and volume), while a large angle indicates a weak negative correlation (e.g., Log *K*_p_ and Log *S*.ESOL). Data points located close to each other in the two-dimensional PC space have similar scores, meaning that they have similar values for the structural and physicochemical properties represented by the variables. Log *S*.ESOL: water solubility; TPSA: topological polar surface area [Å^2^]; MW: molecular weight [g/mol]; BBI: Bertz branching index; SNar: Narumi simple topological index [log function]; Log *K*_p_: skin permeation [cm/s]; volume: molecular volume [Å^3^]. For plot (a) and (b)—antibacterial activity is marked as “+++” for values <16 μg/mL, “++” for values 32–64 μg/mL, “+” for values 128–256 μg/mL and “–” for values ≥512 μg/mL. For plot (c) and (d)—cytotoxicity assay is marked as “+++” for cytotoxic effect starting at 50 μg/mL, “++” for cytotoxic effect starting at 100 μg/mL, “+” for cytotoxic effect starting at 150 μg/mL and “–” for no cytotoxic effect in any of the studied concentrations.

Analyzing the results of PCA conducted on cytotoxic activity (specifically, keratinocytes and fibroblasts) yields similar findings (Figure 6c,d). Peptidomimetic derivatives, when projected onto the PCs, reveal an interesting pattern: those with the lowest cytotoxicity (>150 μg/mL) are notably distant from other derivatives and predominantly situated on the left side of the biplot, exemplified by A-197, A-198, A-209, and A-220. Further examination of the biplot (Figure 6c,d) shows that peptidomimetic derivatives with the highest cytotoxic activity (i.e., having cytotoxicity effect starting at 50 μg/mL for keratinocytes/fibroblasts) are predominantly located on the right side of the biplot, as exemplified by A-164 and A-184. To better understand the mechanistic relationships between the biological activity of peptidomimetic derivatives and their structural and physicochemical properties, an analysis of normalized factor loadings was performed (Figure S2). These loadings quantify the correlation between the original variables and the PCs, providing a measure of how strongly each variable contributes to a given PC. Following Malinowski’s rule, only loadings with absolute values equal to or greater than 0.7 are considered statistically significant and meaningful for interpretation. As shown in Figure S2, the variables that contribute most significantly to PC1 include MW, volume, BBI, SNar, synthetic accessibility, ZM1 V, ON0 V, and ON1 V, as well as DBI, Log *S*.ESOL, and area. Among them, all variables except Log *S*.ESOL are positively correlated with PC1. This implies that peptidomimetic derivatives with low PC1 scores (located on the left side of the PCA biplot) have low values for these variables, whereas derivatives with high PC1 scores (located on the right side) are characterized by higher values for the same variables. Conversely, Log *S*.ESOL, which has a negative loading for PC1, indicates that derivatives located on the left side of the biplot are associated with relatively high water solubility. Moving along the *X*-axis (PC1), the water solubility of these derivatives decreases as their PC1 values increase. Thus, the positioning of peptidomimetic derivatives along PC1 provides important insights into their structural and physicochemical properties. Specifically, inactive derivatives located on the left side of the biplot typically exhibit low molecular weight, branching, surface area, and volume combined with high water solubility. Conversely, the right-hand peptidomimetic derivatives show the opposite trend, with higher values for these parameters and lower solubility. The interpretation of PC2 is less straightforward. PC2 is strongly negatively correlated with Log *K*_p_ and positively correlated with TPSA. As shown in Figure S2, derivatives with varying antibacterial or cytotoxic activities are distributed across both low and high PC2 values. This relatively low discrimination between peptidomimetic derivatives with different biological activities along PC2 can be attributed to the limited variance (13%) explained by this component. Consequently, PC2 contributes less to the variance and provides limited discrimination between derivatives with different biological activities, underscoring the dominant role of PC1 in capturing variance related to molecular weight, branching, surface area, volume, and water solubility.

Due to the slightly different classification of biological activity employed in the cell viability assay, intriguing observations arise from interpreting the PCA biplot presented in Figure 7a,b. As the PCA analysis was conducted within the same space of structural features and physicochemical properties of peptidomimetic derivatives as previous PCA analyses, the positioning of data points (i.e., compounds) in the space defined by the first two PCs remained essentially unchanged. Notably, the marker color changes, indicating the biological activity of the analyzed derivatives. Upon closer examination of Figure 7a,b, it becomes evident that the majority of the studied peptidomimetic derivatives classified within the categories “0,” “+”, “++”, or “+++” are situated on the left side of the plot, while those categorized as “–” are positioned on the right side of the PCA biplot. It is noteworthy that, in derivatives exhibiting biological activity in the cell viability assay, a discernible upward trend in the intensity of biological activity is observable when progressing toward higher PC1 values (along the *X*-axis), indicating reduced water solubility and increased size (i.e., molecular weight, branching, and volume). Furthermore, it is notable that most of the analyzed peptide derivatives that demonstrated high antibacterial and cytotoxic activity were found to be inactive or have negative influence in the cell viability assay, falling into the class labeled “–”. Examples of such compounds include compounds A-164, A-165, A-176, A-179, A-184, and A-210. This observation was further confirmed by two-way hierarchical cluster analysis (2D-HCA).

**Figure 7:**
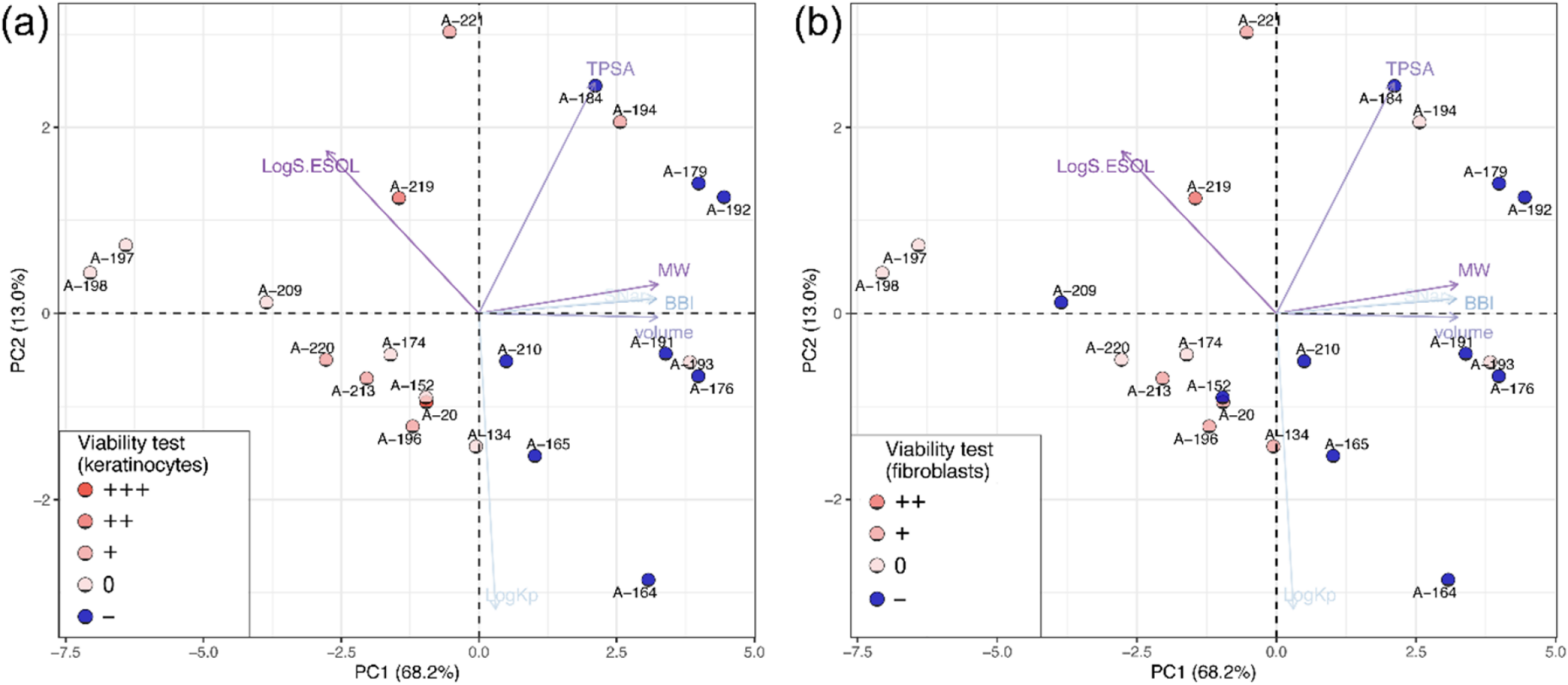
PCA biplot: (a) cytotoxicity assay in human keratinocyte cell line and (b) cytotoxicity on human fibroblast cells. The PCA biplot simultaneously displays the principal component (PC) values of the peptidomimetic derivatives (represented as data points) and the loadings of the explanatory variables (represented as vectors). The length and transparency of the vector represent the variance explained by the variable and its contribution to the PCs, respectively. Variables with the greatest influence on a particular PC are shown as the longest vectors with the most intense coloring. The angles between the vectors indicate correlations: a small angle indicates a strong positive correlation (e.g., MW, BBI, SNar, and volume), while a large angle indicates a weak negative correlation (e.g., Log *K*_p_ and Log *S*.ESOL). Data points located close to each other in the two-dimensional PC space have similar scores, meaning that they have similar values for the structural and physicochemical properties represented by the variables. Log *S*.ESOL: water solubility; TPSA: topological polar surface area [Å^2^]; MW: molecular weight [g/mol]; BBI: Bertz branching index; SNar: Narumi simple topological index [log function]; Log *K*_p_: skin permeation [cm/s]; volume: Molecular volume [Å^3^]. For plots (a) and (b), viability is marked as effect at concentration of 10 μg/mL: “+++” for strong effect, “++” for medium effect, “+” for low effect, “0”—for no effect, and “–” negative effect.

The two-way hierarchical cluster analysis (2D-HCA) in Figure 8 highlights the relationships between the structural and physicochemical properties of peptidomimetic derivatives and their biological activity. Peptidomimetics with similar chemical characteristics cluster together, suggesting that shared structural features result in comparable bioactivity. Higher antibacterial activity appears linked to a large topological polar surface area (TPSA) and specific topological indices. Branching indices (BBI, DBI) influence activity, with highly branched compounds interacting differently with bacterial or human cells. Additionally, a larger energy gap (GAP) correlates with lower biological activity, while high log *P* (Log *S*.ESOL) and low chemical potential may increase cytotoxicity due to enhanced membrane permeability.

**Figure 8:**
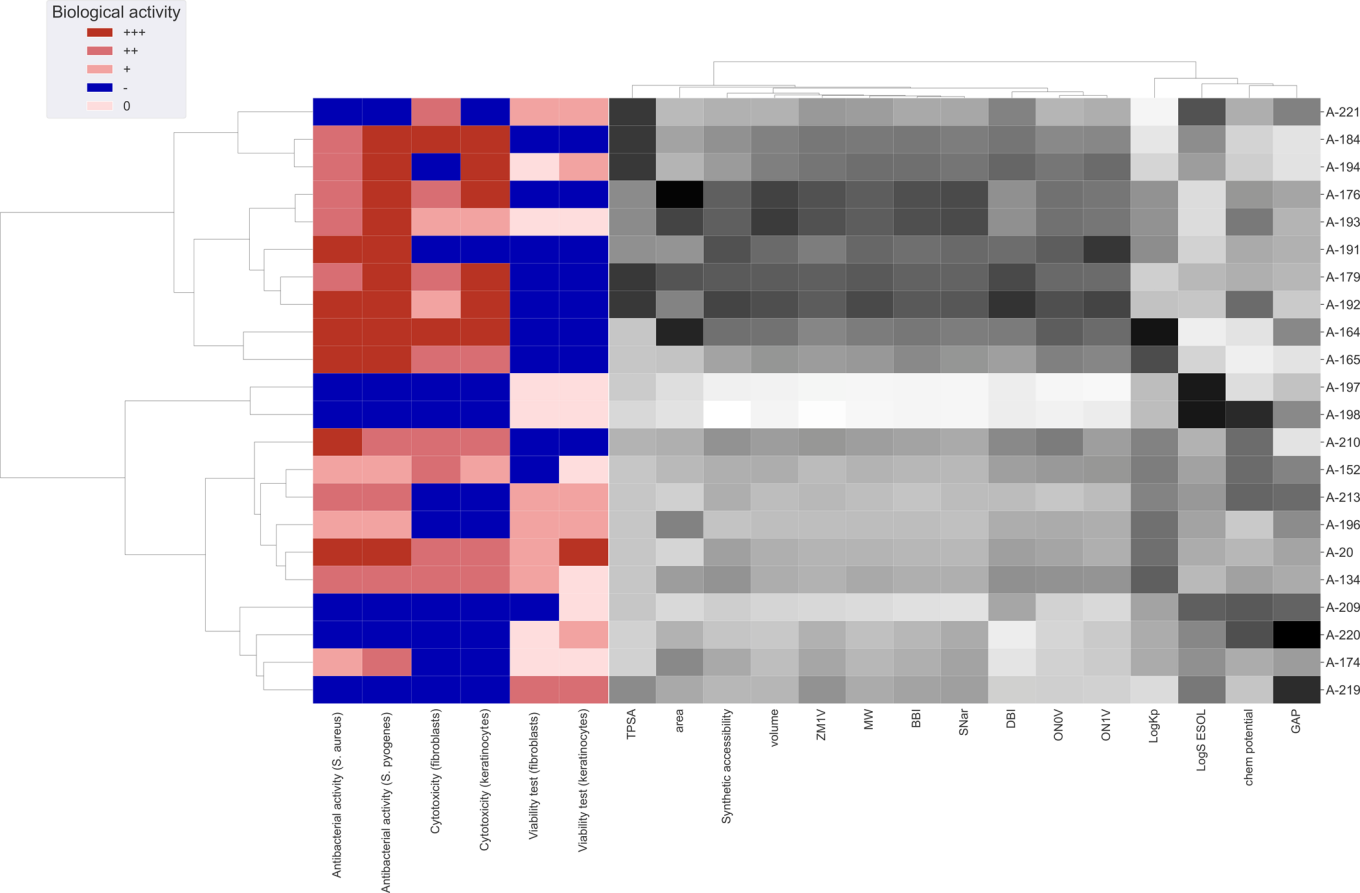
Two-way hierarchical cluster analysis (2D-HCA) with peptidomimetic derivatives on the *Y* axis and their structural features and physicochemical properties on the X-axis. In the dendrograms, branches reflect the level of similarity among variables/observations: shorter branches denote higher similarity, while longer ones indicate greater dissimilarity. TPSA: topological polar surface area [Å^2^]; area: solvent accessible surface area [Å^2^]; synthetic accessibility; volume: molecular volume [Å^3^]; ZM1 V: first Zagreb index by valence vertex degrees; MW: molecular weight [g/mol]; BBI: Bertz branching index; SNar: Narumi simple topological index [log function]; DBI: Dragon branching index; ON0 V and ON1 V: overall modified Zagreb index of order 0 and 1 by valence vertex degrees; Log *K*_p_: skin permeation [cm/s]; Log *S*.ESOL: water solubility; chem.potential: chemical potential [eV] and GAP: the energy difference between the highest occupied molecular orbital and the lowest unoccupied molecular orbital [eV].

A 2D-HCA in Figure 8 confirmed that A-197 and A-198, which boast the highest water solubility and the slightest degree of branching, are notably distant from other peptidomimetic derivatives. This suggests that they differ significantly from the rest of the analyzed compounds. Following closely behind in terms of dissimilarity is A-221, attributed to its remarkably low skin permeation (Log *K*_p_) value. The most potent compound should ideally exhibit high activity in the first two columns of Figure 8 (indicated by red color), low cytotoxicity in the next two columns (indicated by blue color), and pro-proliferative effects in the viability test (indicated as pale pink). A-191 appears to be the most active against bacteria with low cytotoxicity and a slight negative impact on cell line proliferation. A-213, A-196, and A-174 show low antimicrobial activity, no cytotoxic effect, and a pro-proliferative impact on the cell lines. Interestingly, A-196 and A-174 share a highly similar physicochemical profile despite differences in biological activity (they exhibit the most similar profile in the gray color range but differ significantly in the red and blue color ranges), implying that minor structural modifications can significantly alter bioactivity. Moreover, A-197 and A-198 stand out due to their pronounced deviation in several physicochemical parameters from the rest of the data set (mainly white color), hinting at unique structural characteristics that may influence their biological effects.

## Discussion

Peptidomimetics, which mimic natural peptides, are gaining attention in antimicrobial research as alternatives to traditional antibiotics. With the rise of multidrug-resistant bacteria, researchers are exploring new sources, including host defense peptides from multicellular organisms. Antimicrobial peptides are promising compounds for combating pathogenic Gram-positive bacteria.^37–41^ While antimicrobial peptides offer high specificity, potency, and safety,^42,43^ their instability in protease-rich environments is a drawback.^44–46^

In this work, we have designed twenty-one potentially active antimicrobial peptidomimetics based on our previous findings.^20–23^ Using various organic synthesis strategies, we obtained different analogues with subtle structural differences to study their impact on activity. Notably, A-20 is active against multiple *S. aureus* strains and *S. pyogenes*, as previously reported in our studies.^22,23^

In order to achieve good solubility of these compounds during the bacterial susceptibility assessment, the stock solutions were prepared with additives. This was performed to prevent precipitation of the peptidomimetic product during the antimicrobial assay. The additives were 5% of ethanol or 10% of DMSO. Both these solvents have been dermatologically investigated^47–50^ and approved for topical administration by the FDA.

The most potent compounds against *S. aureus* were A-192 and A-164, followed by A-165 and A-191. All four compounds seemed to be active against most of the Gram-positive bacteria studied in this paper. All these compounds include a more sterically hindered moiety in the P4 position than A-20. A-191 and A-192 both contain arginine in the P2 position, differing only in that A-191 has lysine in the P1 position while A-192 has homoarginine. Additional active compounds were A-134, A-176, A-179, A-193, and A-210. Interestingly, despite the minor structural differences between A-192 and A-193, their antimicrobial activity differs significantly. The key distinction lies in the P2 position, where A-192 contains arginine and A-193 has histidine. Despite maintaining the net charge of the compound, these changes influence the overall activity. A study found that substituting arginine with histidine can sometimes maintain certain functions when replacing arginine, though it often leads to reduced efficacy or altered activity due to the unique properties and roles of arginine in various biological processes.^51,52^ This might occur due to the environmental conditions of the bacterial medium. BHI broth maintains a pH of 7.4, where arginine remains in its cationic form, while histidine loses its cationic charge and is almost deprotonated under these conditions.

Compounds A-191 and A-192 exhibited the lowest MIC values against *S. pyogenes* in this study, with A-164 following closely behind. These three compounds share a phenylcinnamic acid residue at the P4 position. Five compounds also exhibited high activity against *S. pyogenes*, with A-165, A-176, A-179, A-193, and A-194 showing activity at two- to three-fold higher concentrations. Once again, all of these compounds contain sterically hindered moieties. A-165 and A-194 share a naphthylacetic acid moiety in the P4 position, while the others contain a phenylcinnamic acid residue in the P4 position. A-184 and A-210 can also be considered active. A-184, in addition to the naphthylacetic acid moiety, possesses two consecutive arginine residues. When analyzing the activity against *S. cohnii*, it is evident that A-192 and A-164 exhibited the lowest MIC values for this strain. Also showing significant activity were A-210, A-179, A-165, and A-134. A-179, which contains a phenylcinnamic acid residue in the P4 position, is similar to A-164, with the exception that it has arginine in the P2 position. Compound A-164 also appeared to be potent against *E. faecalis*, with A-210 showing activity at a concentration one-fold higher. A-210 exhibited activity against *S. schleiferi*, which also demonstrated a high susceptibility to A-164. Additionally, A-191 and A-193 showed antimicrobial activity against this strain. The next bacterial strain investigated in this study was *S. agalactiae*. The most active compound was found to be A-164, followed by A-194. Other compounds exhibiting activity included A-134, A-165, A-192, A-193 and A-210. A sterically hindered moiety is also present in A-165, A-192 and A-193. A-134 features homoarginine at the P1 position. Compound A-210 differs from the original compound only by the presence of a methyl group on the aromatic ring in the ortho position, of the P1 position. It is hard to determine without structural and ligand-donor studies why such slight changes may have such an impact on the activity of a particular molecule. It is well-known in literature that, e.g., addition or removal of a methyl group from the backbone of cyclic peptides can drastically affect the impact on binding affinity,^53^ while methylation of an amide bond, which disrupts the potential for hydrogen bonding, can change the activity.^54^ *S. intermedius* seemed to be more susceptible to the studied compounds. The only ones with no effect were A-197, A-198, A-209, A-219 and A-220. All of these compounds have very drastic changes in the structure, relative to A-20. Both A-197 and A-198 lack one of the aromatic rings, A-197 is missing the whole cinnamoyl moiety (P4 position), while A-198 lacks the whole carboxyloxycarbonyl moiety (P1 position). A-209 is characterized by the absence of an aromatic ring within the cinnamoyl moiety at the P4 position. In contrast, both A-219 and A-220 lack the 1,2-diamine functional group at the P3 position, instead featuring a valine residue in this region. Among the examined peptidomimetics, the most active compound against *S. mutans* was A-213. It differs from A-20 in lacking a CH_3_ group in 1,2-diamine, resulting in reduced branching (from isopropyl to an ethyl substituent). In addition to A-213, A-194 also exhibited activity against *S. mutans*, with a potency similar to A-20. A-194 contains a sterically hindered moiety in the P4 position and features homoarginine instead of arginine in the P1 position. Additionally, it has arginine in the P2 position instead of leucine. Currently, the infections caused by *S. mutans* are treated with chlorhexidine and silver nanoparticles^55^ and some AMPs targeting this bacterium have also been identified.^56^

Despite some peptidomimetics showing MIC values as low as 256 μg/mL, this concentration was still too high to be considered indicative of meaningful activity against any of the studied Gram-negative bacterial (*E. coli*, *P. vulgaris*, *K. pneumoniae*, *P. aeruginosa*—used as a representative of Gram-negative bacteria) strains. The difference in activity between Gram-negative and Gram-positive bacteria may be attributed to distinct structural and compositional differences. Antimicrobial compounds that exhibit selective activity against Gram-positive bacteria, such as vancomycin,^57^ bacitracin,^58^ and daptomycin,^59^ function primarily by targeting structural components of a Gram-positive cell wall. These bacteria possess a thick peptidoglycan layer, enriched with teichoic acids, which readily attracts the cationic nature of antimicrobial peptides. In contrast, Gram-negative bacteria are inherently resistant to many of these agents due to their additional outer membrane, which serves as a permeability barrier and lacks the high-density peptidoglycan structure found in Gram-positive bacteria. Given the cationic nature of our peptidomimetics, it is reasonable to hypothesize that their antimicrobial activity is associated with membrane disruption. However, our previous attempts to confirm this mechanism were unsuccessful.^23^ To gain deeper insights into the mode of action of these peptidomimetics, more advanced microbiological models should be employed.

Given that peptides are prone to degradation by proteolytic enzymes^60,61^ structural modifications are usually introduced to enhance their stability in enzyme-rich fluids.^62^ In our research stability of peptidomimetics in aqueous solutions was assessed, as peptides often tend to precipitate or degrade in solution.^63^ All synthesized peptidomimetics demonstrated stability in water, and the majority exhibited resistance to proteolytic degradation in human plasma. Notably, among all the peptidomimetics tested, A-196, A-219, A-220, and A-221—those with the fewest modifications compared to natural peptides—showed signs of degradation after 24 h of incubation.

The most potent compounds with the highest antimicrobial activity in this study were A-164, A-165, A-191, and A-192. Both A-191 and A-192 contain arginine in the P2 position, an insertion intended to enhance solubility compared to that of A-164. A-164 exhibits a higher hydrophobicity, as indicated by the percentage of acetonitrile required for its elution from the column. Computational ADMET predictions classified A-164 as either poorly soluble or insoluble, depending on the algorithm applied. A-165 appears to be even more hydrophobic than A-164, as evidenced by its longer elution time. However, ADMET predictions classified it as either moderately soluble or insoluble. A-164 and A-165 have the highest calculated lipophilicity due to additional aromatic rings at the P4 position. A-192 is more hydrophobic than A-191, with a moderate hydrophobicity level (% AcCN is 48.7, while the value for A-20 is 53.3). This shift in hydrophobicity relative to that of A-164 is attributed to the substitution of leucine with arginine at the P2 position. Additionally, the presence of an extra −CH_2_– group in the P1 position (arginine to homoarginine) and the incorporation of an additional aromatic ring (cinnamoyl moiety to phenylcinnamoyl moiety) relative to A-20 would be expected to increase hydrophobicity. However, certain intrinsic molecular interactions may influence shifts in solubility in ways not solely dictated by structural modifications.^64–66^ Alternatively, the substitution of leucine with arginine alone may be sufficient to enhance the molecule’s solubility, despite the aforementioned modifications. The hydrophobicity of the compounds appears to be an important factor in their activity, which might be linked to their mechanism of action. The least hydrophobic compounds, namely, A-197, A-198 (lacking certain aromatic rings), A-220, and A-219 (based on both theoretical and experimental data), seem to be completely inactive, as evident from the PCA analysis. In contrast, the most active compounds are positioned on opposite sides of the graph, exhibiting poor solubility but high activity. It is readily noticed that the additional charge (from +1 to +2) in compounds like A-174 (histidine in place of leucine) and A-176 (arginine in place of leucine) decreased the lipophilicity in comparison to A-164.

An additional verification of experimental data and additional information was retrieved by the ADMET theoretical tool. The computational approach is widely known and utilized.^67–71^ In addition to the aforementioned lipophilicity, parameters such as water solubility, gastrointestinal (GI) absorption, P-glycoprotein substrate, skin permeation, BBB permeation, potential of cytochrome inhibition/substrate enzymes, total clearance, renal OCT2 substrate, toxicity, LD50, hepatotoxicity, and skin sensitization were calculated for the investigated molecules.

The gastrointestinal (GI) absorption of the presented compounds was low, and oral administration is not a suitable administration method. All the presented compounds were likely to be substrates for P-gp, which is not so uncommon in antibiotics, e.g., clarithromycin is a substrate for both cytochrome p4503A4 and P-gp.^72^ However, these properties can be improved by putting the compound into a formulation, e.g., into lipids,^73^ administrating it with a P-glycoprotein ATPase inhibitor (e.g., curcumin),^74^ or adding a probiotic which secretes such an inhibitor and boosts absorption.^75^ All of the studied compounds seemed to have low skin permeability. While many antibiotics exhibit poor skin permeation values,^76,77^ their permeability can be altered by choosing the right formulation or by the addition of a penetration enhancer.^76,78–81^ Low skin permeation poses a challenge for topical administration when ulcer treatment. Moreover, none of the studied compounds were predicted to pass the BBB, which is not uncommon, as penicillin’s penetration is as low as 1% in an uninflamed organism.^82^ However, certain antibiotics, such as ampicillin and amoxicillin, can cross the barrier even in absence of inflammation.^83^ The next calculated parameter was VD. Almost all the compounds in this study are considered to be distributed primarily in plasma rather than in tissue, with only A-198 and A-165 exceeding negative values, and three compounds (A-134, A-176, and A-193) potentially exhibiting moderate distribution. The VD values for vancomycin range from −0.398 to 0 log L/kg^84^, and for gentamicin, they range from −0.699 to −0.432 log L/kg.^85^ The results showed that none of the designed compounds are predicted to be substrates of CYP2D6; however, all were likely to be substrates of CYP3A4. None of the compounds were predicted to inhibit the CYP1A2, CYP2C19, and CYP2D6 isoforms, while only two (A-174 and A-220) are likely inhibitors of the CYP3A4 isoform. Nine compounds (A-174, A-179, A-191, A-192, A-194, A-196, A-209, A-210, and A-213) are unlikely to inhibit the CYP2C9 isoform. The metabolism of antimicrobial compounds by CYP enzymes can result in their conversion into active or inactive metabolites, potentially altering their efficacy. Inhibition of CYP enzymes can reduce the metabolism of coadministered drugs, thus increasing plasma concentration and posing a higher risk of toxicity or adverse effects. Vancomycin is an antibiotic that is neither a substrate nor an inhibitor of CYP enzymes, and is primarily cleared by renal function.^86^

Six compounds (A-164, A-179, A-192, A-197, A-210, and A-221) are predicted to be slowly eliminated from the body in total drug clearance, while compounds A-174, A-198, and A-220 exhibit the highest rates of elimination. This implies that these compounds may require more frequent administration or formulation for controlled release. None of the listed compounds are likely substrates for OCT2, which is important when administered with other drugs. Additionally, none of the compounds in this study exhibited mutagenic potential, which might indicate that they are potentially noncarcinogenic. Almost all of the compounds are predicted to be potentially hepatotoxic, with the exception of A-197 and A-198. Many well-known antibiotics, such as amoxicillin and tetracyclines, also present this side effect.^87,88^ Skin sensitization is another critical safety consideration for products applied dermally, as it can lead to allergic dermatitis. None of the tested compounds appeared to potentially irritate the skin, which is crucial for topical administration.

In this study, LDH tests were employed to evaluate the membrane integrity of cells treated with various peptidomimetic compounds, allowing for a comprehensive analysis of their safety profiles. The results from the LDH assays revealed differential cytotoxic effects, underscoring the importance of this assay in the early stages of peptidomimetic drug development. The cytotoxicity levels were studies in concentrations of 50, 100, and 150 μg/mL, which are mostly above the level of MIC values. A-174, A-191, A-197, and A-213 seem to exert no or minimal cytotoxicity toward both cell lines. Only A-191, whose MIC value for *S. aureus* of 16 μg/mL exerts little cytotoxicity at higher concentrations, though this fades with decreasing concentration. Compound A-164, whose MIC value is against *S. aureus* is 8 μg/mL and shows a high cell toxicity profile at concentrations 6-fold higher than its MIC value. It is not uncommon for antibiotics to possess cytotoxic properties, for example, it happens in the case of fluoroquinolones and aminoglycosides^89^ and in the case of topical ointments containing polymixin B, bacitracin, neomycin, and gentamicin.^90^ The results showed that A-192, which seems to be the most potent antimicrobial compound, shows high cytotoxicity toward keratinocytes, though its cytotoxicity toward fibroblasts seems to be much lower. Keratinocytes are cells that are primarily found in the epidermis, whereas fibroblasts are hidden in connective tissue. While both cell types can exhibit sensitivity to chemicals, keratinocytes are generally more fragile than fibroblasts and more responsive to topical chemical exposures.^91^ A-165, A-219, and A-221 are cytotoxic only for fibroblasts and exert a noncytotoxic effect on keratinocytes. A-220 demonstrated consistent cytotoxicity at a level of 10% over all of the concentrations. Compounds A-193, A-194, A-196, A-198, and A-209 become toxic to both cell lines at concentrations of 150 μg/mL. A-210 begins to be cytotoxic at a concentration of 100 μg/mL. The cytotoxicity of A-152 is similar to that of A-20. A-134, A-176, A-179, and A-184 are toxic at all concentrations. The incubation time of the cell with peptidomimetic was prolonged to 48 h due to the long-term use in the potential treatment of wounds with the applied antimicrobial compound. The topical administration of peptidomimetics can avoid strong side effects such as nephrotoxicity or ototoxicity; however, the cytotoxicity to skin cells is very important. The administration of antimicrobial agents for a limited duration, despite their cytotoxic potential, can be clinically advantageous, particularly when targeting multidrug-resistant pathogens such as MRSA. Notable examples of toxic antibiotics utilized in clinical settings include polymyxin E (colistin),^92,93^ chloramphenicol,^94^ vancomycin,^95^ and linezolid.^96,97^ These agents are associated with significant adverse effects, including nephrotoxicity, neurotoxicity, aplastic anemia, and bone marrow suppression. While chloramphenicol is rarely employed due to its hematological toxicity, it remains a viable option in the management of life-threatening infections, such as bacterial meningitis, in cases where alternative treatments are unavailable.^94,98^

Peptides and peptidomimetics are capable of exerting dual actions, such as both antimicrobial and antiviral effects (as in the case of A-20 ^99^) or antimicrobial and pro-proliferative effects.^100^ The safety profile of A-20 has previously been reported,^22^ demonstrating no impact on the proliferation of primary human keratinocytes at concentrations up to 50 μg/mL, and no induction of inflammatory responses or allergic reactions. The cell proliferation assay was specifically designed to evaluate whether the compounds could independently stimulate cell proliferation. To eliminate background and accurately assess the direct effects of the tested compounds, serum-free conditions were employed throughout the experiment with FBS serving as a positive control.

Many peptidomimetics exhibit dose-dependent effects, with antiproliferative activity at higher concentrations and pro-proliferative activity at lower concentrations, as seen with A-134. Some peptidomimetics, such as A-152, require extended exposure times to exert their effects, with notable activity observed after only 72 h. Among the tested compounds, A-219 exhibited the strongest pro-proliferative effect on both cell lines across all concentrations, demonstrating a dose-dependent relationship. A-174, although showing no significant impact on cells, exhibited a subtle pro-proliferative effect, while A-213 displayed a positive impact after 72 h at low concentrations. Compounds A-164, A-165, A-176, A-179, A-184, A-191, A-192, A-209, and A-210 demonstrated antiproliferative effects across nearly all concentrations, with increased activity at higher concentrations. A-193 and A-194 displayed an antiproliferative effect at high concentrations; however, after 72 h, a strong pro-proliferative effect was observed in fibroblasts. Their antimicrobial efficacy was noted at concentrations between 32 and 64 μg/mL, which falls outside the pro-proliferative range, suggesting a safer profile for wound application. A-196 showed a slight pro-proliferative effect at low concentrations but became cytotoxic at higher concentrations. An antiproliferative effect was noted for A-197 and A-198 only at high concentrations after 72 h of incubation in both cell lines, while no such effect was observed at 48 h. A-220 exhibited a slight pro-proliferative effect on keratinocytes after 72 h at low concentrations, while A-221 exhibited a pro-proliferative effect on fibroblasts after 48 h at low concentrations but displayed mild toxicity at higher concentrations.

Compounds with the most potent antimicrobial activity, such as A-164, A-165, and A-192 (considering only activity against *S. aureus*), exhibited antiproliferative effects on the tested cell lines. However, their impact on cell proliferation decreases to 50% at 50 μg/mL and remains at this level up to the maximum tested concentration of 150 μg/mL. Other compounds with notable antibacterial activities include A-176, A-179, A-191, and A-193. A-191 nearly completely inhibits proliferation at higher concentrations, whereas A-179 progressively suppresses proliferation as its concentration increases. A-176 and A-193 exhibit similar behavior to A-164, A-165, and A-192 in terms of their effects on proliferation, with a decline in cell viability as the concentration of peptidomimetic increases. The final groups of compounds with antimicrobial activity to be considered include A-184, A-194, A-210, and A-213. For A-184 and A-210, proliferation decreases with increasing concentrations of peptidomimetics, reaching 50% at the highest tested concentration. A-213 shows antiproliferative effects at high concentrations, but at lower concentrations, it stimulates proliferation by 20–30%. A-194 displays pro-proliferative effects at low concentrations, but at concentrations exceeding 100 μg/mL, it demonstrates antiproliferative properties.

Many empirical and computational SAR methods are being applied to investigate a series of compounds in order to establish which structural (chemical structure, secondary structure, and flexibility) or physicochemical (hydrophobicity and net charge) properties are fundamental to their biological activity. In this case, a PCA analysis was used. This allowed us to discover how structural features and physicochemical properties are responsible for the molecular biological activity. The biological results had to be simplified in order to be analyzed. The antimicrobial activity of the peptidomimetics was calculated only by two bacterial strains, namely *S. aureus* and *S. pyogenes,* divided into four classes. A biological assay was gathered in 4 classes for cytotoxicity and 5 classes for the proliferation assay, distinguishing only cytotoxicity and pro-proliferative effects for both cell lines and without distinguishing the time over which the incubation was conducted. The features exerting the most significant influence on the separation of peptidomimetic derivatives are water solubility, molecular weight (MW), degree of branching (BBI and SNar), and the compound’s volume. Inactive compounds (such as A-197 and A-198) exhibit notably higher water solubility and lower molecular mass, branching, and volume compared to active compounds (e.g., A-192 and A-193). This observation aligns with the general trend indicating that reduced water solubility and increased size (i.e., molecular weight, branching, and volume) correlate with heightened efficacy against *S. aureus* and *S. pyogenes*. Furthermore, it is important to note that most of the analyzed peptidomimetic derivatives, despite showing strong antibacterial activity, exerted some cytotoxic activity at high concentrations. Examples of these compounds include compounds A-164, A-165, A-176, A-179, A-184, and A-210. This finding was further validated by a two-way hierarchical cluster analysis (2D-HCA), which confirmed these findings. Intriguingly, certain peptidomimetic derivatives, such as A-176, A-179, A-184, A-193, and A-194, exhibited greater antibacterial activity against *S. pyogenes* than *S. aureus* upon exposure. In other words, *S. pyogenes* demonstrated heightened sensitivity to these studied derivatives. The variance in responses to peptidomimetic derivative exposure is hard to analyze when the molecular target, as well as the mechanism of action of these compounds, remains unknown. Moreover, slight disparities in sensitivity were observed in the cytotoxic activity and viability tests. The human keratinocyte cell line exhibited greater sensitivity in both scenarios than did human fibroblast cells.

## Conclusions

This study explores the potential of structurally modified peptidomimetic analogues of cystatin C as promising antimicrobial agents against Gram-positive pathogens, particularly, *S. aureus* and *S. pyogenes*. Through rational design and synthesis of novel compounds, we demonstrate how subtle modifications in molecular architecture—including changes in charge, hydrophobicity, and steric hindrance—can significantly influence antimicrobial efficacy and cytocompatibility. Two key compounds exhibited potent antimicrobial activity with acceptable cytotoxicity levels, while others showed selective effects depending on concentration and exposure time. However, challenges such as solubility issues and limited efficacy against Gram-negative bacteria underscore the need for further optimization of these compounds. Importantly, most derivatives were stable in aqueous solutions and human plasma, with low predicted mutagenic and skin sensitization potential. ADMET predictions also supported the potential for topical use due to low systemic absorption and favorable physicochemical properties. The theoretical studies revealed a correlation between structural features, offering insight into key parameters driving efficacy. These findings lay a strong foundation for further preclinical development and formulation strategies aimed at localized treatment of drug-resistant Gram-positive infections, particularly in wound or skin-associated contexts. Overall, our results demonstrate that peptidomimetic optimization guided by both experimental and computational approaches can yield lead compounds with therapeutic potential and encourage continued exploration of peptide-inspired compounds in antimicrobial drug development. Future studies should focus on investigating the molecular mechanism underlying the antimicrobial activity of these compounds.

## Supplementary Material



## Notes

Symbols and definitions of all ADME-related parameters and molecular descriptors used for exploratory data analysis (Table S1); exemplary HPLC analysis of the stability of peptidomimetics (Figure S1); plot of the normalized factor loadings (Figure S2); % of acetonitrile required for elution from the column and theoretical and experimental masses measured by mass spectrometry of synthesized compounds (Table S2); results of microbiological assay for all 22 compounds for eight Gram-positive and four Gram-negative bacteria (Table S3) (PDF)
