# Supplementary material for: Role of Structural Modifications in Peptidomimetic Compounds as Potential Antimicrobial Agents against Staphylococcus aureus and Streptococcus pyogenes: Balancing Bioavailability, Safety, and Antimicrobial Activity
Source: ACS Omega. 2025 Jul 22;10(30):33435–60. doi: 10.1021/acsomega.5c03775 (PMC12332667; doi:10.1021/acsomega.5c03775)
Supplement: Supplementary file 1 [file ao5c03775_si_001.pdf]

Role of Structural Modifications in Peptidomimetic Compounds as Potential Antimicrobial Agents Against *Staphylococcus aureus* and *Streptococcus pyogenes:* Balancing Bioavailability, Safety, and Antimicrobial Activity

Maria Dzierżyńska[1*], Justyna Sawicka[1], Katarzyna Łada[1], Agnieszka Gajewicz-Skrętna[2], Milena Deptuła[3], Alexey Chernobrovkin[4, 5], Aneta Pogorzelska[6], Anders Grubb[7], Roman A. Zubarev[4], Michał Pikuła[3], Franciszek Kasprzykowski[1], Sylwia Rodziewicz-Motowidło[1]

[1]Department of Biomedical Chemistry, Faculty of Chemistry, University of Gdańsk, Wita Stwosza 63, 80-308, Gdańsk, Poland

[2]Department of Molecular Biotechnology, Mossakowski Medical Research Institute Polish Academy of Science, Wita Stwosza 63, 80 -308 Gdańsk, Poland

[3]Department of Physical Chemistry, Faculty of Chemistry, Gdańsk University of Technology, Narutowicza 11/12, 80-233, Gdańsk, Poland

[4]Laboratory of Tissue Engineering and Regenerative Medicine, Division of Embryology, Medical University of Gdańsk, Dębinki 1, 80-211, Gdańsk, Poland

[5]Department of Medical Biochemistry and Biophysics, Karolinska Institutet, Solnavägen 9, 17177, Stockholm, Sweden

[6]Pelago Bioscience AB, Scheelesväg 1, 17165, Solna, Sweden

[7]Department of Organic Chemistry, Faculty of Pharmacy, Medical University of Gdańsk, Hallera 107, 80-416, Gdańsk, Poland

[8]Department of Clinical Chemistry, Skåne University Hospital, Lund University, Klinikgatan 19, 22185, Lund Sweden

*Corresponding author: maria.dzierzynska@ug.edu.pl

Table S1. Symbols and definitions of all ADME-related parameters and molecular descriptors used for exploratory data analysis

ID	Name	Description	Software	Included in the analysis?
**ADMET PARAMETERS**				
1	MW	Molecular weight	SwissADME	YES
2	Num, heavy atoms	Number of heavy atoms	SwissADME	-
3	Num, arom, heavy atoms	Number of aromatic heavy atoms	SwissADME	-
4	Fraction Csp3	Fraction csp3	SwissADME	-
5	Num, rotatable bonds	Number of rotatable bonds	SwissADME	-
6	Num, H-bond acceptors	Number of H-bond acceptors	SwissADME	-
7	Num, H-bond donors	Number of H-bond donors	SwissADME	-
8	Molar refractivity	Molar refractivity	SwissADME	-
9	TPSA	Topological polar surface area	SwissADME	YES
10	Log Po/w	Lipophilicity; n-octanol/water partition coefficient	pkCSMY	-
11	LogS (ESOL)	Water solubility	pkCSMY	YES
12	Water solubility	Water solubility [mg/ml]	pkCSMY	-
13	Water solubility	Water solubility [mol/l]	pkCSMY	-
14	LogKp	Skin permeability constant logKp (cm/h)	pkCSMY	YES
15	Synthetic accessibility	Synthetic Accessibility Score	pkCSMY	YES

16	Charge	Charge	pkCSMY	-
**QUANTUM - MECHANICAL DESCRIPTORS**				
17	HOF	Standard heat of formation [kcal/mol]	MOPAC2016	-
18	area	Solvent accessible surface [$Å^2$]	MOPAC2016	YES
19	volume	Molecular volume [$Å^3$]	MOPAC2016	YES
20	dipole	Total dipole moment [Debye]	MOPAC2016	-
21	HOMO	Energy of the highest occupied molecular orbital [eV]	MOPAC2016	-
22	LUMO	Energy of the lowest unoccupied molecular orbital [eV]	MOPAC2016	-
23	IP	Ionization potential [eV]	MOPAC2016	-
24	EA	Electron affinity [eV]	MOPAC2016	-
25	η	Chemical hardness [eV]	MOPAC2016	-
26	S	Chemical softness [eV]	MOPAC2016	-
27	chem.potential	Chemical potential [eV]	MOPAC2016	YES
28	χ^c	Mulliken's electronegativity [eV]	MOPAC2016	-
29	ω	Electrophilicity [eV]	MOPAC2016	-
30	E_g	HOMO-LUMO energy gap [eV]	MOPAC2016	YES
**CONSTITUTIONAL INDICES**				
31	Sv	Sum of atomic van der Waals volumes (scaled on Carbon atom)	DRAGON7	-
32	Se	Sum of atomic Sanderson electronegativities (scaled on Carbon atom)	DRAGON7	-
33	Sp	Sum of atomic polarizabilities (scaled on Carbon atom)	DRAGON7	-
34	Si	Sum of first ionization potentials (scaled on Carbon atom)	DRAGON7	-
35	Mv	Mean atomic van der Waals volume (scaled on Carbon atom)	DRAGON7	-
36	Me	Mean atomic Sanderson electronegativity (scaled on Carbon atom)	DRAGON7	-
37	Mp	Mean atomic polarizability (scaled on Carbon atom)	DRAGON7	-
38	Mi	Mean first ionization potential (scaled on Carbon atom)	DRAGON7	-
39	nAT	Number of atoms	DRAGON7	-
40	nSK	Number of non-H atoms	DRAGON7	-
41	nBT	Number of bonds	DRAGON7	-
42	nBO	Number of non-H bonds	DRAGON7	-
43	nBM	Number of multiple bonds	DRAGON7	-
44	SCBO	Sum of conventional bond orders (H-depleted)	DRAGON7	-
45	RBN	Number of rotatable bonds	DRAGON7	-
46	RBF	Rotatable bond fraction	DRAGON7	-
47	nDB	Number of double bonds	DRAGON7	-
48	nAB	Number of aromatic bonds	DRAGON7	-
49	nH	Number of Hydrogen atoms	DRAGON7	-
50	nC	Number of Carbon atoms	DRAGON7	-
51	nN	Number of Nitrogen atoms	DRAGON7	-

52	nO	Number of Oxygen atoms	DRAGON7	-
53	nHet	Number of heteroatoms	DRAGON7	-
54	H%	Percentage of H atoms	DRAGON7	-
55	C%	Percentage of C atoms	DRAGON7	-
56	N%	Percentage of N atoms	DRAGON7	-
57	O%	Percentage of O atoms	DRAGON7	-
58	nCsp3	Number of sp3 hybridized Carbon atoms	DRAGON7	-
59	nCsp2	Number of sp2 hybridized Carbon atoms	DRAGON7	-
**RING DESCRIPTORS**				
60	nCIC	Number of rings (cyclomatic number)	DRAGON7	-
61	nCIR	Number of circuits	DRAGON7	-
62	TRS	Total ring size	DRAGON7	-
63	Rperim	Ring perimeter	DRAGON7	-
64	MCD	Molecular cyclized degree	DRAGON7	-
65	RCI	Ring complexity index	DRAGON7	-
66	NRS	Number of ring systems	DRAGON7	-
67	NNRS	Normalized number of ring systems	DRAGON7	-
68	nBnz	Number of benzene-like rings	DRAGON7	-
69	ARR	Aromatic ratio	DRAGON7	-
**TOPOLOGICAL INDICES**				
70	ZM1	First Zagreb index	DRAGON7	-
71	ZM1V	First Zagreb index by valence vertex degrees	DRAGON7	YES
72	ZM1Kup	First Zagreb index by Kupchik vertex degrees	DRAGON7	-
73	ZM1Mad	First Zagreb index by Madan vertex degrees	DRAGON7	-
74	ZM1Per	First Zagreb index by perturbation vertex degrees	DRAGON7	-
75	ZM1MulPer	First Zagreb index by multiplicative perturbation vertex degrees	DRAGON7	-
76	ZM2	Second Zagreb index	DRAGON7	-
77	ZM2V	Second Zagreb index by valence vertex degrees	DRAGON7	-
78	ZM2Kup	Second Zagreb index by Kupchik vertex degrees	DRAGON7	-
79	ZM2Mad	Second Zagreb index by Madan vertex degrees	DRAGON7	-
80	ZM2Per	Second Zagreb index by perturbation vertex degrees	DRAGON7	-
81	ZM2MulPer	Second Zagreb index by multiplicative perturbation vertex degrees	DRAGON7	-
82	ON0	Overall modified Zagreb index of order 0	DRAGON7	-
83	ON0V	Overall modified Zagreb index of order 0 by valence vertex degrees	DRAGON7	YES
84	ON1	Overall modified Zagreb index of order 1	DRAGON7	-
85	ON1V	Overall modified Zagreb index of order 1 by valence vertex degrees	DRAGON7	YES
86	Qindex	Quadratic index	DRAGON7	-
87	BBI	Bertz branching index	DRAGON7	YES

88	DBI	Dragon branching index	DRAGON7	YES
89	SNar	Narumi simple topological index (log function)	DRAGON7	YES
90	HNar	Narumi harmonic topological index	DRAGON7	-
91	GNar	Narumi geometric topological index	DRAGON7	-
92	Xt	Total structure connectivity index	DRAGON7	-
93	Dz	Pogliani index	DRAGON7	-
94	Ram	Ramification index	DRAGON7	-
95	BLI	Kier benzene-likeliness index	DRAGON7	-
96	Pol	Polarity number	DRAGON7	-
97	LPRS	Log of product of row sums (PRS)	DRAGON7	-
98	MSD	Mean square distance index (Balaban)	DRAGON7	-
99	SPI	Superpendentic index	DRAGON7	-
100	PJI2	2D Petitjean shape index	DRAGON7	-
101	ECC	Eccentricity	DRAGON7	-
102	AECC	Average eccentricity	DRAGON7	-
103	DECC	Eccentric	DRAGON7	-
104	MDDD	Mean distance degree deviation	DRAGON7	-
105	UNIP	Unipolarity	DRAGON7	-
106	CENT	Centralization	DRAGON7	-
107	VAR	Variation	DRAGON7	-
108	ICR	Radial centric information index	DRAGON7	-
109	SMTI	Schultz Molecular Topological Index (MTI)	DRAGON7	-
110	SMTIV	Schultz Molecular Topological Index by valence vertex degrees	DRAGON7	-
111	GMTI	Gutman Molecular Topological Index	DRAGON7	-
112	GMTIV	Gutman Molecular Topological Index by valence vertex degrees	DRAGON7	-
113	Xu	Xu index	DRAGON7	-
114	CSI	Eccentric connectivity index	DRAGON7	-
115	Wap	All-path Wiener index	DRAGON7	-
116	S1K	1-path Kier alpha-modified shape index	DRAGON7	-
117	S2K	2-path Kier alpha-modified shape index	DRAGON7	-
118	S3K	3-path Kier alpha-modified shape index	DRAGON7	-
119	PHI	Kier flexibility index	DRAGON7	-
120	PW2	Path/walk 2 - Randic shape index	DRAGON7	-
121	PW3	Path/walk 3 - Randic shape index	DRAGON7	-
122	PW4	Path/walk 4 - Randic shape index	DRAGON7	-
123	PW5	Path/walk 5 - Randic shape index	DRAGON7	-
124	MAXDN	Maximal electrotopological negative variation	DRAGON7	-
125	MAXDP	Maximal electrotopological positive variation	DRAGON7	-

126	DELS	Molecular electrotopological variation	DRAGON7	-
127	TIE	E-state topological parameter	DRAGON7	-
128	Psi_i_s	Intrinsic state pseudoconnectivity index - type S	DRAGON7	-
129	Psi_i_A	Intrinsic state pseudoconnectivity index - type S average	DRAGON7	-
130	Psi_i_0	Intrinsic state pseudoconnectivity index - type 0	DRAGON7	-
131	Psi_i_1	Intrinsic state pseudoconnectivity index - type 1	DRAGON7	-
132	Psi_i_t	Intrinsic state pseudoconnectivity index - type T	DRAGON7	-
133	Psi_i_0d	Intrinsic state pseudoconnectivity index - type 0d	DRAGON7	-
134	Psi_i_1d	Intrinsic state pseudoconnectivity index - type 1d	DRAGON7	-
135	Psi_i_1s	Intrinsic state pseudoconnectivity index - type 1s	DRAGON7	-
136	Psi_e_A	Electrotopological state pseudoconnectivity index - type S average	DRAGON7	-
137	Psi_e_0	Electrotopological state pseudoconnectivity index - type 0	DRAGON7	-
138	Psi_e_1	Electrotopological state pseudoconnectivity index - type 1	DRAGON7	-
139	Psi_e_t	Electrotopological state pseudoconnectivity index - type T	DRAGON7	-
140	Psi_e_0d	Electrotopological state pseudoconnectivity index - type 0d	DRAGON7	-
141	Psi_e_1d	Electrotopological state pseudoconnectivity index - type 1d	DRAGON7	-
142	Psi_e_1s	Electrotopological state pseudoconnectivity index - type 1s	DRAGON7	-
143	BAC	Balaban centric index	DRAGON7	-
144	LOC	Lopping centric index	DRAGON7	-
145	XMOD	Modified Randic index	DRAGON7	-
146	RDCHI	Reciprocal distance sum Randic-like index	DRAGON7	-
147	RDSQ	Reciprocal distance sum inverse Randic-like index	DRAGON7	-
148	X1Kup	Kupchik connectivity index	DRAGON7	-
149	X1Mad	Connectivity topochemical index	DRAGON7	-
150	X1Per	Perturbation connectivity index	DRAGON7	-
151	X1MulPer	Multiplicative perturbation connectivity index	DRAGON7	-
152	ISIZ	Information index on molecular size	DRAGON7	-
153	IAC	Total information index on atomic composition	DRAGON7	-
154	AAC	Mean information index on atomic composition	DRAGON7	-
155	IDE	Mean information content on the distance equality	DRAGON7	-
156	IDM	Mean information content on the distance magnitude	DRAGON7	-
157	IDDE	Mean information content on the distance degree equality	DRAGON7	-
158	IDDM	Mean information content on the distance degree magnitude	DRAGON7	-
159	IDET	Total information content on the distance equality	DRAGON7	-
160	IDMT	Total information content on the distance magnitude	DRAGON7	-
161	IVDE	Mean information content on the vertex degree equality	DRAGON7	-
162	IVDM	Mean information content on the vertex degree magnitude	DRAGON7	-
163	S0K	Kier symmetry index	DRAGON7	-

164	HVcpx	Graph vertex complexity index	DRAGON7	-
165	HDcpx	Graph distance complexity index (log function)	DRAGON7	-
166	Uindex	Balaban U index	DRAGON7	-
167	Vindex	Balaban V index	DRAGON7	-
168	Xindex	Balaban X index	DRAGON7	-
169	Yindex	Balaban Y index	DRAGON7	-

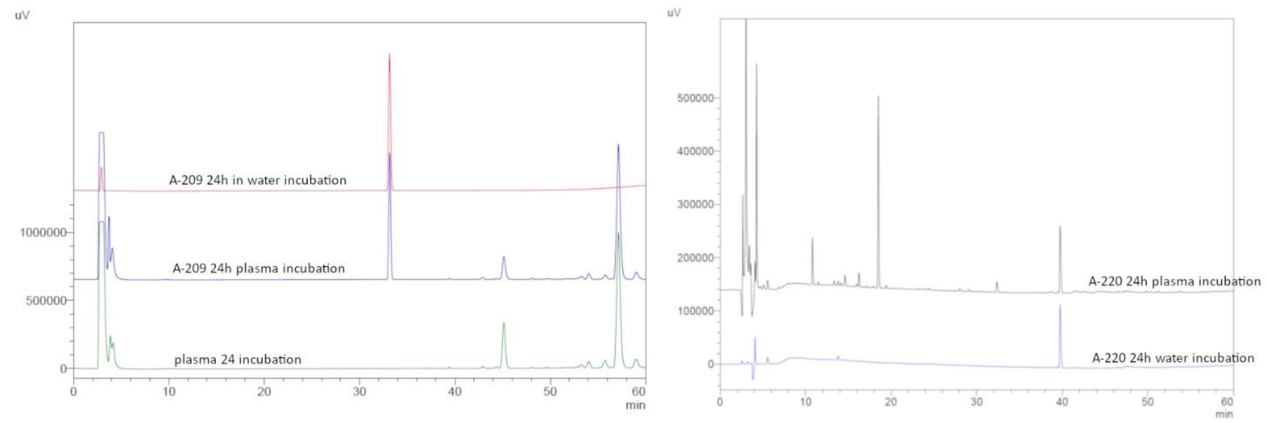

Figure 1S. Exemplary HPLC analysis of peptidomimetics: left panel - comparison chromatograms of A-209 after 24h of incubation in water in 37˚C, after 24h incubation in plasma and plasma alone after 24h (detection method ELSD-LT II), right panel – comparison chromatograms of A-220 after 24h incubation in plasma and after 24h of incubation in water (detection method UV-Vis λ=223 nm)

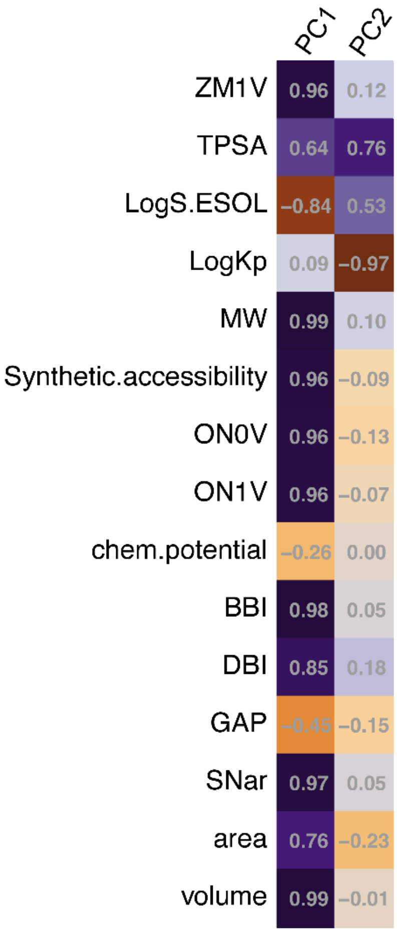

Figure S2. The plot of the normalized factor loadings. TPSA: topological polar surface area [Å^2]; area: solvent accessible surface area [Å^2]; synthetic accessibility; volume: Molecular volume [Å^3]; ZM1V: first Zagreb index by valence vertex degrees; MW: molecular weight [g/mol]; BBI: Bertz branching index; SNar: Narumi simple topological index [log function]; DBI: Dragon branching index; ON0V & ON1V: overall modified Zagreb index of order 0 and 1 by valence vertex degrees; LogKp: skin permeation [cm/s]; LogS.ESOL: water solubility; chem.potential: chemical potential [eV] and GAP: the energy difference between highest occupied molecular orbital and lowest unoccupied molecular orbital [eV].

Table S2. % of acetonitrile required for elution from the column and theoretical and experimental masses measured by mass spectrometry of synthesized compounds.

No	Code	% AcCN	Monoisotopic mass [Da]	Experimental mass $[M+H]^+$ [Da]	Experimental mass $[M+2H]^+$ [Da]
**1**	A-20	53.3	635.38	636.29	318.65
**2**	A-134	56.6	649.40	650.40	Not present
**3**	A-152	56.5	637.40	638.26	319.64
**4**	A-164	62.9	711.42	712.39	356.71
**5**	A-165	65.3	673.40	674.36	337.68
**6**	A-174	42.9	631.35	632.35	316.61
**7**	A-176	50.4	735.39	736.39	368.68
**8**	A-179	49.8	754.43	755.40	378.18
**9**	A-184	45.3	716.41	717.45	359.17
**10**	A-191	44.8	726.42	727.38	364.19
**11**	A-192	48.7	768.44	768.40	385.21
**12**	A-193	49.0	749.40	750.32	375.67
**13**	A-194	44.8	730.43	731.49	366.17
**14**	A-196	62.3	623.38	624.34	Not present
**15**	A-197	42.5	505.38	506.30	253.66
**16**	A-198	40.0	503.36	504.32	252.67
**17**	A-209	49.6	573.36	574.36	Not present
**18**	A-210	57.4	649.40	650.39	Not present
**19**	A-213	54.6	621.36	622.37	Not present

**20**	A-219	42.6	647.35	648.31	324.64
**21**	A-220	42.4	619.35	620.23	310.62
**22**	A-221	43.9	666.40	667.38	334.15

Table S3. Results of microbiological assay for all 22 compounds for eight Gram-positive and four Gram-negative bacteria.

		MIC [µg/ml]/MBC [µg/ml]																																			
		Gram-Positive Bacteria																								Gram-Negative Bacteria											
		S. aureus ATCC 43300			*S. pyogenes* ATCC 19615			*S. cohnii* c.i.			*E. faecalis* c.i.			*S. schleiferi* c.i.			*S. agalactiae* ATCC 27956			*S. intemrdius* c.i.			*S. mutans* c.i.			*E. coli* ATCC 25922			*P. vulgaris* ATCC 6896			*K. pneumoniae* ATCC 700603			*P. aeruginosa* ATCC 10145		
Water additive		*H_2O*	*EtOH*	*DMSO*	*H_2O*	*EtOH*	*DMSO*	*H_2O*	*EtOH*	*DMSO*	*H_2O*	*EtOH*	*DMSO*	*H_2O*	*EtOH*	*DMSO*	*H_2O*	*EtOH*	*DMSO*	*H_2O*	*EtOH*	*DMSO*	*H_2O*	*EtOH*	*DMSO*	*H_2O*	*EtOH*	*DMSO*	*H_2O*	*EtOH*	*DMSO*	*H_2O*	*EtOH*	*DMSO*	*H_2O*	*EtOH*	*DMSO*
1	A-20	16/32	16/32	16/32	8/16	8/16	8/8	128/64	64/64	64/64	128/256	128/256	128/256	16/16	16/16	8/8	128/256	64/128	32/64	32/64	32/64	16/32	16/n.d.	16/ n.d.	16/ n.d.	256/512	256/512	256/512	>512	>512	>512	>512	>512	>512	>512	>512	>512
2	A-134	32/n.d	32/64	64/128	32/n.d.	16/32	32/16	n.d.	32/32	32/32	n.d.	512/512	256/512	n.d.	>512	>512	n.d.	32/64	32/64	n.d.	16/16	32/32	n.d.	>512	512/>512	n.d.	>512	>512	n.d.	>512	>512	n.d.	>512	>512	n.d.	>512	>512
3	A-152	128/256	64/128	512/512	128/256	64/126	128/256	n.d.	128/512	128/512	n.d.	512/512	512/>512	n.d.	>512	>512	n.d.	128/256	128/128	n.d.	64/64	64/64	n.d.	>512	>512	n.d.	>512	>512	n.d.	>512	>512	n.d.	>512	>512	n.d.	>512	>512
4	A-164	8/16	8/16	8/16	8/16	4/8	8/16	n.d.	32/64	16/32	32/64	32/64	16/32	8/16	4/8	16/32	8/16	4/8	16/32	16/32	8/16	4/8	>512	>512	>512	>512	>512	>512	>512	>512	>512	>512	>512	>512	>512	>512	>512
5	A-165	16/32	16/16	16/64	16/32	16/16	16/32	n.d.	32/32	64/128	n.d.	64/128	64/128	n.d.	>512	>512	n.d.	32/64	32/32	n.d.	16/32	32/32	n.d.	512/>512	>512	>512	>512	>512	>512	>512	>512	>512	>512	>512	>512	>512	512
6	A-174	256/512	512/512	512/512	64/256	64/256	128/256	256/256	128/256	128/256	>512	>512	>512	>512	512/512	>512	512/512	256/512	512/512	256/512	256/512	128/256	>512	>512	>512	>512	>512	>512	>512	512/>512	>512	>512	>512	>512	>512	512/>512	>512
7	A-176	32/64	32/64	32/128	16/32	16/32	16/64	64/128	128/128	128/128	512/512	256/512	>512	16/64	16/32	64/64	128/128	128/128	128/128	64/128	64/128	128/128	32/64	32/64	64/128	256/256	512	>512	>512	>512	>512	>512	>512	>512	256	256/256	256/256
8	A-179	32/32	32/32	16/32	16/32	8/32	16/32	16/32	32/64	32/32	128/128	128/256	256/256	128/128	128/256	128/128	64/128	64/64	64/128	16/32	16/16	16/32	>512	>512	>512	>512	>512	>512	>512	>512	>512	512	>512	>512	256	>512	>512
9	A-184	64/64	64/128	64/64	32/64	16/32	16/16	64/128	128/256	128/256	>512	>512	>512	32/32	16/32	16/32	256/512	256/512	256/512	64/128	32/64	64/128	32/64	32/64	64/128	512/>512	64/128	>512	>512	>512	>512	>512	>512	>512	128/256	64/128	128/128
10	A-191	32/32	16/32	16/32	4/4	4/8	4/8	64/64	64/64	64/64	512/>512	>512	512/>512	8/32	8/32	8/32	128/128	128/128	128/128	32/64	32/64	32/64	64/128	32/64	32/64	512/>512	512/>512	512/>512	512/>512	512/>512	>512	>512	512/>512	>512	64/256	64/128	64/256
11	A-192	4/4	16/16	16/16	8/8	4/8	8/8	16/32	16/32	32/32	>512	256/512	>512	256/256	256/256	256/256	32/64	32/64	64/64	8/16	8/16	8/16	>512	>512	>512	>512	>512	256//512	>512	>512	>512	>512	>512	>512	64/256	>512	64/128
12	A-193	32/32	32/32	32/32	8/16	8/16	8/16	64/128	64/128	128/256	256/512	128/256	512	8/32	8/32	4/8	64/128	32/64	128/128	16/32	16/32	32/64	64/128	8/16	8/16	256/256	256/512	>512	>512	>512	>512	>512	>512	>512	>512	>512	512
13	A-194	64/64	16/32	64/64	16/32	16/32	32/64	64/128	64/128	64/128	>512	512/512	>512	8/16	32/64	16/32	32/32	16/16	64/64	32/64	32/64	32/64	16/32	16/32	16/32	512	>512	512	>512	512/>512	>512	128/256	64	128/128	>512	>512	>512
14	A-196	128/256	64/128	128/256	64/128	128/256	128/256	64/128	128/256	128/256	>512	512/>512	>512	16/16	16/32	16/16	64/128	64/128	64/128	32/128	64/128	64/128	128/256	64/128	32/64	>512	>512	>512	>512	>512	>512	>512	>512	>512	>512	>512	>512
15	A-197	>512	>512	>512	>512	>512	>512	>512	>512	>512	>512	>512	>512	256	256	256	>512	>512	>512	>512	>512	>512	256/512	128/256	64/128	>512	>512	>512	>512	>512	>512	>512	>512	>512	>512	>512	>512
16	A-198	>512	>512	>512	>512	>512	>512	512/>512	512/512	512/512	>512	>512	>512	>512	>512	>512	>512	>512	>512	>512	>512	>512	>512	>512	512/>512	>512	>512	>512	>512	>512	>512	>512	>512	>512	>512	>512	>512
17	A-209	>512	>512	>512	>512	>512	>512	>512	>512	>512	>512	>512	>512	256/512	256/512	256/512	>512	>512	>512	>512	>512	>512	128/256	128/256	128/256	>512	>512	>512	>512	>512	>512	>512	>512	>512	>512	>512	>512
18	A-210	32/64	16/32	32/64	32/64	32/64	32/64	64/128	32/64	32/64	64/256	32/256	64/256	4/8	4/8	8/16	16/64	32/64	16/32	16/16	16/16	16/16	>512	>512	>512	>512	>512	>512	128/256	128	128/256	>512	>512	>512	512	256/512	512
19	A-213	64/128	64/128	64/128	64/128	64/128	64/128	64/128	64/128	64/128	256/256	256/256	256/256	32/32	32/32	16/32	64/64	64/128	64/128	32/64	32/64	32/64	4/8	4/8	4/8	256/256	256/256	256/512	>512	>512	>512	>512	512	512	512	256/256	256/512
20	A-219	>512	>512	>512	512/512	512/512	512/512	>512	>512	>512	>512	>512	>512	>512	>512	>512	>512	>512	>512	>512	>512	>512	>512	>512	>512	>512	>512	>512	>512	>512	>512	>512	>512	>512	512/>512	512/>512	256/512
21	A-220	512/512	512/512	512/512	512/>512	512/512	128/256	256/512	256/512	256/512	>512	>512	>512	>512	>512	>512	512/512	512/512	512/512	512/512	512/512	512/512	256/512	>512	>512	512/512	512/512	>512	>512	>512	>512	>512	>512	>512	512/>512	512/>512	512/>512
22	A-221	512/512	256/512	>512	128/256	128/256	512/>512	128/256	128/256	128/256	>512	>512	>512	>512	>512	>512	256/512	256/512	256/512	128/128	128/256	128/256	>512	>512	>512	>512	>512	>512	>512	>512	>512	>512	>512	>512	128/256	64/128	32/64
